# Supplementary material for: Comprehensive targeting of resistance to inhibition of RTK signaling pathways by using glucocorticoids
Source: Nat Commun. 2021 Dec 1;12:7014. doi: 10.1038/s41467-021-27276-7 (PMC8636603; doi:10.1038/s41467-021-27276-7)
Supplement: Supplementary file 1 — Supplementary Information [file 41467_2021_27276_MOESM1_ESM.pdf]

Supplementery Table 1

Gong et al.

A

Cytokines	SuperFamily	Erl vs Ctrl	Enb+Erl vs Enb	Tha+Erl vs Tha	PRDN+Erl vs PRDN
TNFSF13B	TNF	12.83	6.621	-9.709	2.641
FAM19A2	Chemokine	9.15	0.787	7.872	-1.173
IFNE	IFN	8.59	7.086	10.350	-6.723
TNF	TNF	8.23	3.121	-0.430	-0.588
TGFB2	TGFB	7.67	3.431	8.916	2.651
TNFSF4	TNF	7.06	4.418	4.919	12.428
IL11	IL6	6.15	10.004	-0.073	-1.908
CNTF	IL6	4.89	7.879	12.877	-4.735
PPBP	Chemokine	4.65	-6.532	-1.882	-5.926
TNFSF8	TNF	4.55	-1.228	2.150	2.408
CCL27	Chemokine	3.91	-7.186	3.886	1.331
CCL2	Chemokine	3.79	9.563	13.332	4.816
CCL5	Chemokine	3.63	-0.362	1.728	4.189
TNFSF14	TNF	3.47	-2.542	5.221	10.855
LTB	TNF	2.72	16.322	6.482	6.822
CCR1	Chemokine	2.71	3.013	1.352	0.362
CLCF1	IL6	2.63	3.261	1.928	3.113
CXCL5	Chemokine	2.12	2.316	4.284	-3.278
OSM	IL6	2.07	-2.775	0.957	-3.174

B A549

Ligand	Receptor	Ctrl	PRDN	Erl	P+E	p	q
NRG1	ERBB2/3	0.00	-1.04	2.24	-1.04	0.003	0.028
EFNA2	EPNA2	0.00	-0.71	1.78	-0.56	0.003	0.028
NRG2	ERBB2/3	0.00	1.62	1.71	1.87	0.472	0.417
HGF	MET/MSP	0.00	-0.94	1.63	-0.31	0.024	0.041
CSF1	CSFR1	0.00	-0.59	1.61	0.12	0.011	0.03
FGF1	FGFR1	0.00	-0.35	1.51	-0.37	0.01	0.03
EFNA1	EPNA1	0.00	-0.16	1.32	0.07	0.012	0.03
GAS6	AXL	0.00	-0.39	1.24	0.00	0.036	0.047
PDGFA	PDGFRA	0.00	0.24	1.24	0.30	0.095	0.104
NRG4	ERBB2/3	0.00	1.05	1.16	0.64	0.095	0.104
COL6A3	DDR1/2	0.00	-0.52	1.14	0.72	0.255	0.236
ANGPT2	TIE2	0.00	0.00	1.12	0.02	0.004	0.028
EFNA5	EPNA5	0.00	-0.44	1.11	0.24	0.014	0.03
VEGFD	VEGFR2/3	0.00	-0.13	1.07	0.20	0.019	0.039
PDGFB	PDGFRB	0.00	-0.44	0.88	-0.97	0.022	0.04
GDNF	RET	0.00	-0.27	0.52	-0.56	0.011	0.03
FGF2	FGFR1	0.00	-0.57	0.46	-0.74	0.007	0.03
NGF	TRKA	0.00	-0.73	0.28	0.21	0.542	0.459
COL4A3	DDR1/2	0.00	0.99	0.27	1.00	0.031	0.046
ANGPT1	TIE1	0.00	-0.02	0.21	-0.03	0.028	0.045
WNT5A	ROR1/2,RYK	0.00	-0.04	0.16	0.09	0.723	0.531
PROS1	DTK	0.00	0.30	0.09	0.21	0.664	0.523
EGF	EGFR	0.00	-0.89	0.01	-1.12	0.009	0.03
COL4A4	DDR1/2	0.00	0.27	-0.15	0.20	0.176	0.176
HBEGF	EGFR	0.00	-2.39	-0.16	-2.16	0.035	0.047
COL5A2	EGFR	0.00	-0.79	-0.30	-0.79	0.054	0.066
BDNF	TRKB	0.00	-0.17	-0.43	0.00	0.256	0.236
EREG	EGFR	0.00	-1.19	-0.51	-0.54	0.617	0.504
VEGFC	VEGFR2/3	0.00	-0.73	-0.52	-0.60	0.8	0.569
NTF3	TRKC	0.00	-1.12	-1.02	-0.81	0.715	0.531
NRG3	ERBB2/3	0.00	-1.37	-2.41	-3.50	0.118	0.124

C HCC827

Ligand	Receptor	Ctrl	PRDN	Erl	P+E	p	q
HGF	MET/MSP	0.00	-0.95	2.18	-1.26	0.0014	0.0219
NRG1	ERBB2/3	0.00	-0.66	1.68	-1.09	0.0062	0.031
GDNF	RET	0.00	-2.24	1.28	-0.35	0.002	0.0219
FGF2	FGFR1	0.00	-1.85	1.26	-0.31	0.0223	0.0433
COL6A3	DDR1/2	0.00	-0.59	1.00	0.39	0.0644	0.0812
COL4A3	DDR1/2	0.00	0.54	0.90	0.43	0.1076	0.1232
EFNA2	EPNA2	0.00	-0.77	0.83	-1.04	0.0272	0.0489
CSF1	CSFR1	0.00	-0.86	0.74	-0.92	0.0165	0.0432
GAS6	AXL	0.00	-1.60	0.59	-0.34	0.0377	0.0559
NGF	TRKA	0.00	0.09	0.54	-0.75	0.0453	0.0634
FGF1	FGFR1	0.00	-2.83	0.40	-2.38	0.0045	0.0284
EFNA1	EPNA1	0.00	-1.18	0.36	-1.10	0.0189	0.0433
HBEGF	EGFR	0.00	-1.96	0.21	-2.00	0.0103	0.0369
ANGPT1	TIE1	0.00	0.58	0.14	0.37	0.5153	0.4329
VEGFD	VEGFR2/3	0.00	-0.04	0.10	-0.07	0.3338	0.3235
COL5A2	EGFR	0.00	-0.62	0.01	-0.09	0.5969	0.4852
PROS1	DTK	0.00	0.09	-0.07	-0.74	0.0079	0.0331
NRG4	ERBB2/3	0.00	0.92	-0.11	-0.49	0.0026	0.0219
EGF	EGFR	0.00	-1.06	-0.11	-1.47	0.0222	0.0433
ANGPT2	TIE2	0.00	-0.76	-0.22	-0.78	0.0516	0.0685
BDNF	TRKB	0.00	-0.76	-0.22	-0.83	0.1212	0.1328
EFNA5	EPNA5	0.00	-0.96	-0.29	-1.16	0.1647	0.1661
EREG	EGFR	0.00	-0.75	-0.29	-0.83	0.0325	0.0512
WNT5A	ROR1/2,RYK	0.00	-0.09	-0.31	-0.43	0.3982	0.3717
VEGFC	VEGFR2/3	0.00	-0.45	-0.33	-0.98	0.0148	0.0432
NTF3	TRKC	0.00	-0.97	-0.42	-0.61	0.5025	0.4329
COL4A4	DDR1/2	0.00	-1.01	-0.61	-0.93	0.0887	0.1065
PDGFA	PDGFRA	0.00	0.03	-0.62	-1.49	0.031	0.0512
PDGFB	PDGFRB	0.00	-0.47	-0.81	-0.77	0.4897	0.4329
NRG2	ERBB2/3	0.00	-1.07	-1.33	-2.47	0.1394	0.1463
NRG3	ERBB2/3	0.00	-1.58	-0.87	-2.63	0.0171	0.0432

Supplementary Table 1 Erlotinib-induced Cytokines; erlotinib upregulates a large number of RTK ligands and most were suppressed by concomitant prednisone. A. From the RNA seq data in Figure 1B, cytokines with z-normalized Gene Set Enrichment Analysis (GSEA) metric scores over 1.96 ($p < 0.05$) at erlotinib versus control were selected to show their GSEA scores in the indicated 4 comparisons. **B-C**. EGFR wt A549 cells and EGFR mutant HCC827 cells were treated with 1 μM or 100 nM erlotinib, with or without 10 μM prednisolone for 3 days. Total RNA was extracted and subjected to real-time PCR for detecting mRNA levels of multiple RTK ligands as listed. Real-time PCR were performed in 3 independent experiments, showing each value and mean ± SEM. The mean values, p-values and q-values of multiple t-tests were summarized in this table. The multiple comparison correction was corrected by two-stage step-up method of Benjamini, Krieger and Yekutieli. The desired False Discovery Rate (FDR) was set as 0.05. The statistical analysis above was performed on Graphpad Prism 9.0.0.

Supplementary Table 2 Gong et al.

RTK Ligand	RTK	Erl vs Ctrl	Enb+Erl Vs Enb	Tha+Erl Vs Tha	PRDN+Erl Vs PRDN
IGF2	Insulin R	11.91	10.35	5.49	8.01
EFNB3	EPHR	11.42	-2.78	6.15	9.69
FGF2	FGFR	8.91	4.28	4.01	0.21
ANGPT1	TIE	8.84	2.67	7.09	-10.04
ANGPT2	TIE	8.49	5.15	14.02	-8.58
COL15A1	DDR	8.40	-8.66	-10.29	0.29
VEGFD	VEGFR	8.07	12.58	2.73	-2.66
EFNB2	EPHR	7.93	5.08	6.56	10.59
COL12A1	DDR	7.55	3.98	5.06	0.82
COL11A1	DDR	6.92	0.00	0.00	-0.56
FGF12	FGFR	6.90	8.51	-2.94	3.84
WNT5A	ROR	6.49	8.00	-1.07	-3.28
FGF18	FGFR	5.89	12.44	-1.72	8.67
COL24A1	DDR	5.81	3.42	-3.01	2.21
KITLG	PDGFR	5.67	3.08	11.71	-3.07
NRG4	ERBB	5.39	5.29	4.25	1.14
TUB	Axl	5.22	2.85	-1.84	4.69
COL6A3	DDR	5.20	6.26	-3.73	-3.41
COL26A1	DDR	4.38	1.39	7.56	-2.96
COL4A4	DDR	4.20	4.66	1.72	-1.66
NRG1	ERBB	4.06	0.33	-0.23	-2.70
COL11A2	DDR	3.98	1.00	7.68	0.44
CSF1	PDGFR	3.59	4.01	5.19	3.44
BTC	EGFR	3.47	-0.11	12.79	5.33
COL4A3	DDR	3.32	4.33	5.02	-3.93
COL6A6	DDR	3.30	-2.82	-4.94	-3.47
HBEGF	EGFR	3.17	-4.14	-3.88	0.39
HGF	HGFR	3.12	2.94	2.39	-7.49
COL10A1	DDR	2.01	1.19	-8.87	7.79

Supplementary Table 2 Erlotinib-induced RTK ligands. From the RNA seq data in Figure 1B, RTK ligands with GSEA z-scores over 1.96 at erlotinib versus control were selected to show their GSEA z-scores in the indicated 4 comparisons. Here we examined the effect on Enbrel (etanercept), thalidomide or prednisolone on erlotinib induced expression of RTK ligands.

Supplementary Figure 1 Gong et al.

A

Erlotinib-induced Pathways	Erl-induced	Enb-block	Tha-block	PRDN-block
Inflammatory Response	111/122up	10%	25%	57%
Receptor tyrosine kinase(RTK)	89/90up	7%	12%	97%
WNT	20/22up	10%	35%	65%
Nuclear Receptor (AR,ER,NR)	11/11up	9%	27%	82%
Notch	9/10up	22%	67%	78%
PI3K-AKT	7/7up	0%	14%	100%
SMAD	5/5up	0%	20%	100%
RHO	8/8up	0%	25%	75%
AURORA	2/2up	0%	0%	100%
BMP	2/2up	0%	0%	100%

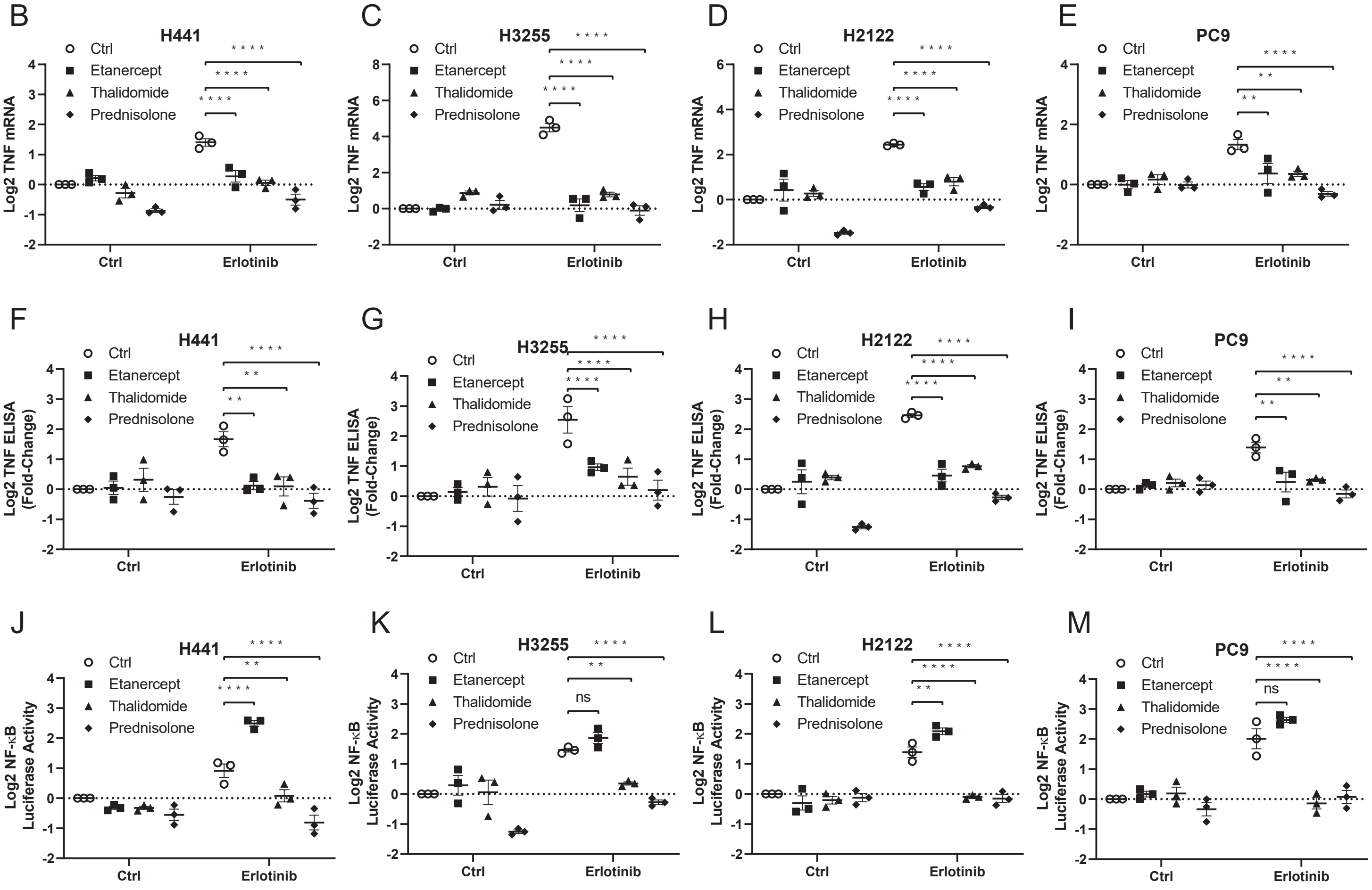

Supplementary Figure 1.

Erlotinib induced pathways and their suppression by three TNF inhibitors.

A. From the A549 RNAseq data shown in Figure 1, around 3000 canonical pathways (c2.cp.v7.2.symbols.gmt) from Gene Set Enrichment Analysis (GSEA) were analyzed in the 4 comparisons. The meaning of 111/122up in erlotinib induced Inflammatory Response is that, among 122 Inflammatory Response Pathways in these 3000 pathways, 111 were found to be induced by erlotinib. The pathway with positive Normalized Enrichment Score (NES) suggests it was upregulated by erlotinib, while negative NES means the pathways can no longer induced by erlotinib. The percentage means the number of pathways blocked by these 3 drugs. For example, prednisolone (PRDN) blocking 57% Inflammatory Response means there are 63 (63/111=57%) Inflammatory Response Pathways with a negative NES in PRDN+Erl versus PRDN. Additional pathways upregulated by erlotinib were blocked by these 3 drugs in different percentages, such as RTK, WNT, Nuclear receptor, etc. **B-M.** TNF mRNA and protein levels, as well as NF-κB activity were examined in additional EGFR wt (H441 and H2122), and mutant (H3255 and PC9) cells. After treatments of 1 μM (H441, H2122) or 10nM (PC9 and H3255) erlotinib in combination with 100 μg/mL etanercept, 10 μM thalidomide, 10 μM prednisolone for 24 hours, RNA was extracted for real-time PCR, and NF-κB activity was determined by luciferase assay. After treatment for 72 hours, cell lysates were tested by ELISA. Experiments were performed in 3 independent times, showing each value and mean ± SEM. *: $p < 0.05$, **: $p < 0.01$, ***: $p < 0.001$, ****: $p<0.0001$, by two-way ANOVA, adjusted by Bonferroni's test. The statistical analysis above was performed on Graphpad Prism 9.0.0.
p=5e-3,3e-4,1e-5;7e-6,4e-7,4e-7;5e-5,7e-5,2e-6;0.005,0.002,4e-5;0.003,0.006,9e-5;7e-7,2e-6,1e-6;2e-5,5e-5,3e-6;0.007,0.004,3e-4;3e-5,0.01,5e-7;5e-6,2e-7;0.01,2e-5,8e-6;3e-6,2e-6

Supplementary Figure 2

Gong et al.

A

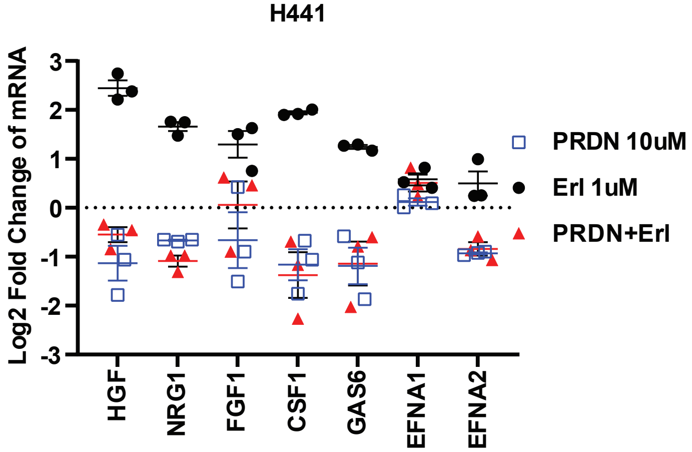

B

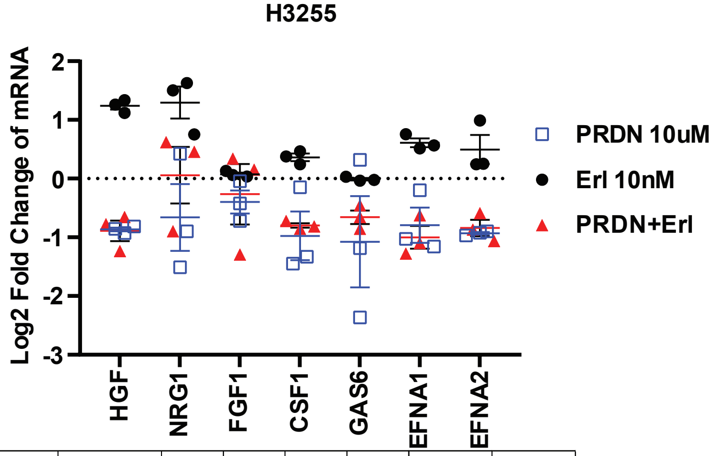

Ligand	Receptor	Ctrl	PRDN	Erl	P+E	p	q
HGF	MET/MSP	0.00	-1.13	2.44	-0.55	0.0037	0.0105
NRG1	ERBB2/3	0.00	-0.67	1.66	-1.09	0.0001	0.0001
FGF1	FGFR1	0.00	-0.66	1.29	0.06	0.0057	0.0272
CSF1	CSFR1	0.00	-1.16	1.94	-1.38	0.0057	0.0219
GAS6	AXL	0.00	-1.19	1.24	-1.14	0.0066	0.0374
EFNA1	EPNA1	0.00	0.12	0.58	0.51	0.1231	0.8206
EFNA2	EPNA2	0.00	-0.93	0.50	-0.84	0.0037	0.0101

Ligand	Receptor	Ctrl	PRDN	Erl	P+E	p	q
HGF	MET/MSP	0.00	-0.87	1.24	-0.89	0.0015	0.0028
NRG1	ERBB2/3	0.00	-0.66	1.29	0.06	0.0051	0.0272
FGF1	FGFR1	0.00	-0.40	0.07	-0.27	0.0892	0.5946
CSF1	CSFR1	0.00	-0.98	0.36	-0.80	0.0008	0.0008
GAS6	AXL	0.00	-1.08	-0.01	-0.66	0.0051	0.0294
EFNA1	EPNA1	0.00	-0.79	0.61	-1.00	0.0051	0.0248
EFNA2	EPNA2	0.00	-0.93	0.50	-0.84	0.0035	0.0101

C

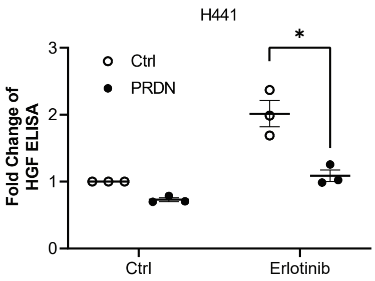

D

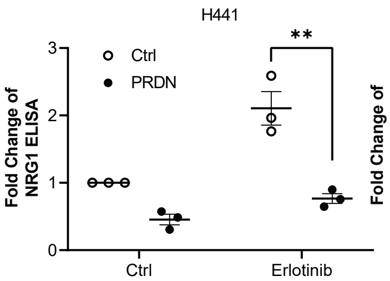

E

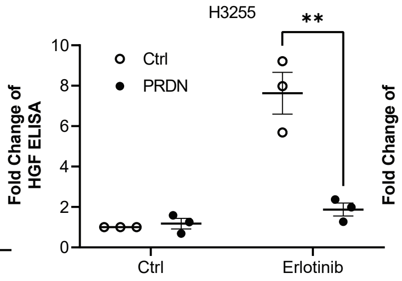

F

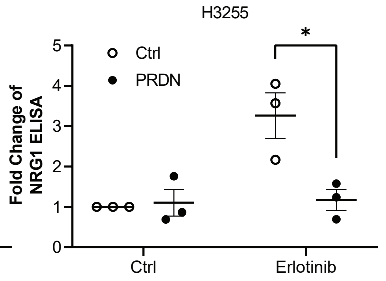

Supplementary Figure 2.

Erlotinib-induced upregulation of RTK ligands and their suppression by prednisone in additional cell lines.

A-B. Some indicated RTK ligands mRNA levels were detected in H441 and H3255 cells, after the indicated treatments for 72 hours. Real-time PCR were performed in 3 independent experiments, showing each value and mean ± SEM. The mean values, p-values and q-values of multiple t-tests were summarized in the table. The multiple comparison correction was corrected by two-stage step-up method of Benjamini, Krieger and Yekutieli. The desired False Discovery Rate (FDR) was set as 0.05. **C-F.** HGF and NRG1 protein levels were tested by ELISA, under the same treatment condition as PCR. 3 independent experiments show each result and mean ± SEM. *: $p < 0.05$, *: $p < 0.01$, by two-way analysis of variance (ANOVA), adjusted by Bonferroni's test. The statistical analysis above was performed on Graphpad Prism 9.0.0. p=0.03,0.006,0.002,0.04.

Supplementary Figure 3 Gong et al.

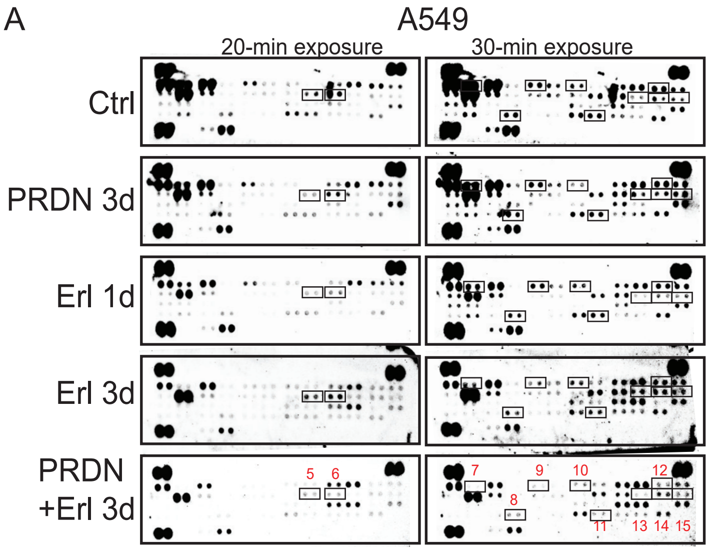

1.MET 2.EGFR 3.ERBB3 4.RYK 5.CSFR 6.RET 7.ERBB2 8.EPHB2
9.FGFR1 10.FGFR3 11.DDR1 12.AXL 13.ROR1 14. ROR2 15.TIE1

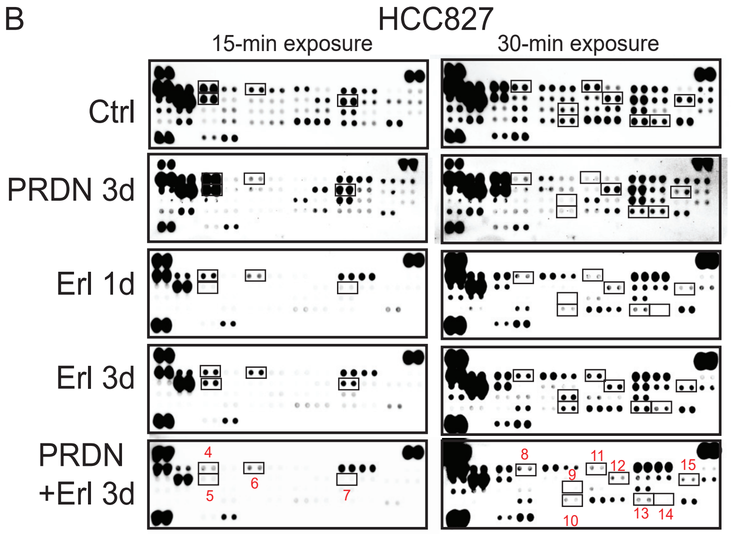

1.EGFR 2.MET 3.ERBB2 4.ERBB3 5.MSP 6.FGFR1 7.RET 8.ERBB4
9.VEGFR2 10.EPHB6 11.FGFR3 12. CSFR 13.DDR2 14. EPHA5 15.ROR2

Supplementary Figure 3.

Additional exposures at extended time points for the RTK array shown in Figure 3

A. Longer exposures of RTK arrays demonstrating the effect of erlotinib on RTK upregulation and their suppression by prednisolone in A549 cells. **B**. A similar experiment done in HCC827 cells.

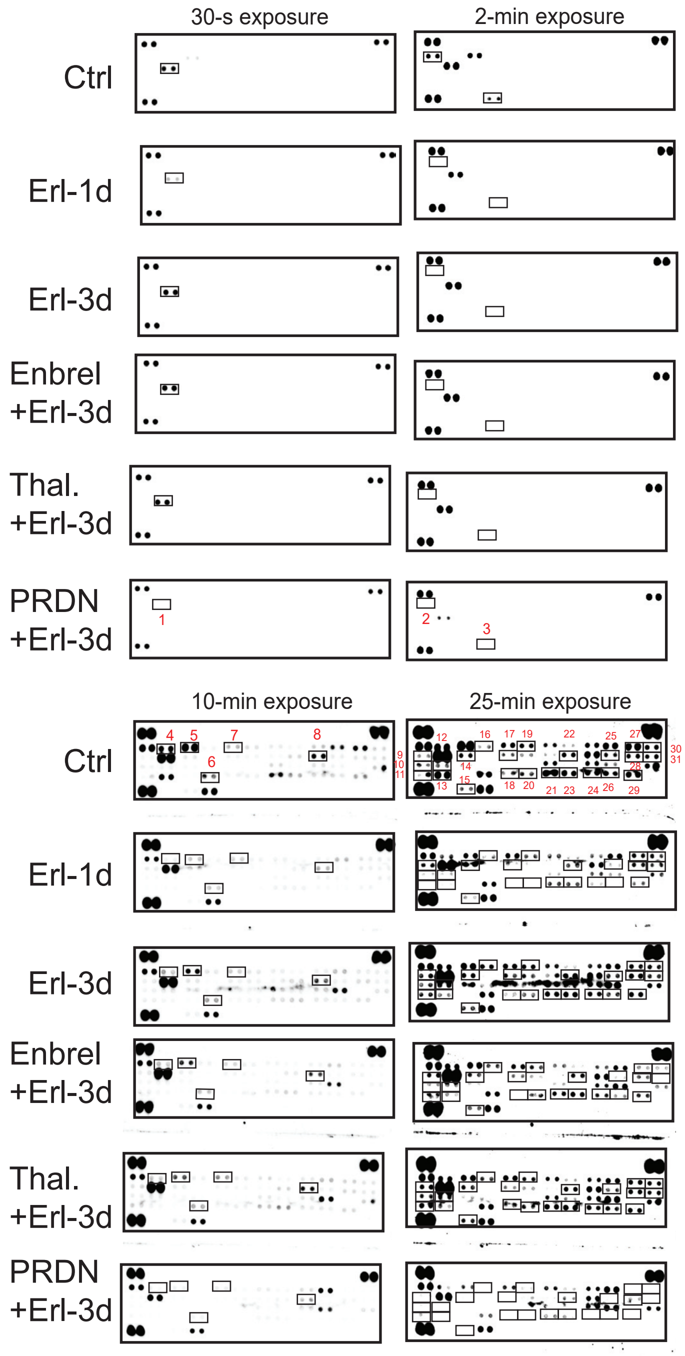

1.MET 2.EGFR 3.RYK 4.ERBB2 5. ERBB3 6.EPHB2 7.FGFR1 8.RET 9.MER 10.TIE2 11.EPHA6 12.TRKA 13.EPHA7 14.MSP 15.EPHB3 16. ERBB4 17. PDGFRB 18. EPHB4 19.FGFR2 20.EPHB6 21.ALK 22.CSFR1 23. DDR1 24.DDR2 25.ROR1 26.EPHA5 27. AXL 28.ROR2 29.EPHA10 30.DTK 31.TIE1

	Ctrl	Erl-1d	Erl-3d	NE-3d	TE-3d	PE-3d
MET	0	-1.05	0.22	0.23	0.20	-2.66
ERBB3	0	-3.59	-2.54	-2.87	-3.09	-4.95
EGFR	0	-1.87	-1.30	-1.35	-1.30	-1.20
RYK	0	-0.92	-1.15	-1.34	-1.22	-1.63
ERBB2	0	-2.27	-1.38	-1.32	-1.19	-2.87
EPHA7	0	-3.74	-2.78	-2.76	-2.56	-4.12
AXL	0	-1.60	-1.80	-2.89	-1.86	-3.98
ALK	0	-2.09	-1.39	-1.71	-1.13	-2.62
RET	0	-0.83	-0.30	-0.61	-0.43	-1.13
EPHA4	0	-1.68	-1.27	-2.39	-0.54	-1.77
IGF1R	0	-0.57	-0.66	-1.22	-0.92	0.49
EPHB2	0	-0.89	-0.40	-0.82	-0.47	-1.96
DDR2	0	-1.98	-0.98	-1.66	-1.00	-3.51
EPHA10	0	-1.35	-0.75	-1.61	-0.94	-2.71
DDR1	0	-1.14	-0.29	-0.57	0.08	-2.06
EPHA5	0	-1.63	-0.56	-1.50	-0.52	-2.43
FGFR1	0	0.19	-0.02	-0.22	0.21	-0.98
INSR	0	-0.49	-0.51	-1.01	-0.77	-0.75
DTK	0	-0.16	-0.35	-1.34	-0.56	-1.92
EPHA1	0	-0.60	0.85	-0.58	-0.02	0.30
ROR2	0	-0.03	0.27	-0.89	-0.16	-0.74
TRKA	0	-0.64	0.78	1.03	0.71	-1.32
TIE1	0	0.02	-0.23	-1.85	-0.33	-1.75
CSF1R	0	0.12	0.88	-0.19	-0.13	-0.92
VEGFR3	0	-1.00	1.45	-1.20	-0.11	-0.64
FGFR2	0	-0.04	0.11	-0.34	-0.48	-1.33
MST1	0	1.08	0.17	-0.13	-0.29	-1.51
ROR1	0	-0.45	0.13	-1.31	-0.59	-0.94
EPHB4	0	-2.76	-0.54	-1.61	0.28	-2.46
EPHB6	0	-1.51	-0.24	0.08	0.33	-2.43
EPHA6	0	-1.52	0.02	0.49	1.00	-0.88
FGFR3	0	0.43	0.15	-0.32	-0.08	-0.38
TIE2	0	-0.73	0.20	-0.12	0.38	-1.14
EPHA2	0	-0.22	2.41	1.61	2.38	1.76
MERTK	0	0.70	0.61	0.80	0.88	-0.66
EPHB3	0	-0.10	0.56	0.54	0.33	-1.05
PDGFRB	0	1.05	0.95	0.31	-0.09	-0.66
KIT	0	0.33	0.94	-0.11	-0.13	
FGFR4	0	0.72	0.17	-0.03	-0.38	-0.76
ERBB4	0	0.93	1.08	0.90	0.45	-1.50
TRKB	0	-0.54	1.52	0.17	0.73	-1.13
FLT3	0	0.33	1.21	-0.62	-0.57	-0.63
EPHA3	0	-2.99	-0.29	-4.54	-1.79	-2.41
EPHB1	0	-3.02	0.05	-0.09	0.23	-1.57
MUSK	0	-0.52	2.30	-0.28	0.82	0.50
TRKC	0	-3.78	1.95	-0.15	1.21	-0.65
PDGFRA	0	2.11	1.66	1.04	0.65	0.34
VEGFR1	0	-3.00	3.88	0.26	1.27	0.36
VEGFR2	0	-1.39	3.51	1.25	1.56	2.27

Color scale: 3, 2, 1, 0, -1, -2, -3, -4

Supplementary Figure 4.

Erlotinib-induced bypass RTK signaling is suppressed by prednisolone but not by (Enbrel) etanercept or thalidomide in RTK arrays

A549 cells were treated with control vehicle for 3 days, 1 μM erlotinib for 1 and 3 days, erlotinib in combination with 100 μg/ml Enbrel (etanercept), 10 μM thalidomide, or 10 μM Prednisolone for 3 days. Six protein samples were subjected to Proteome Profiler Human Phospho-RTK Array as described in the Methods.

Supplementary Figure 5

Gong et al.

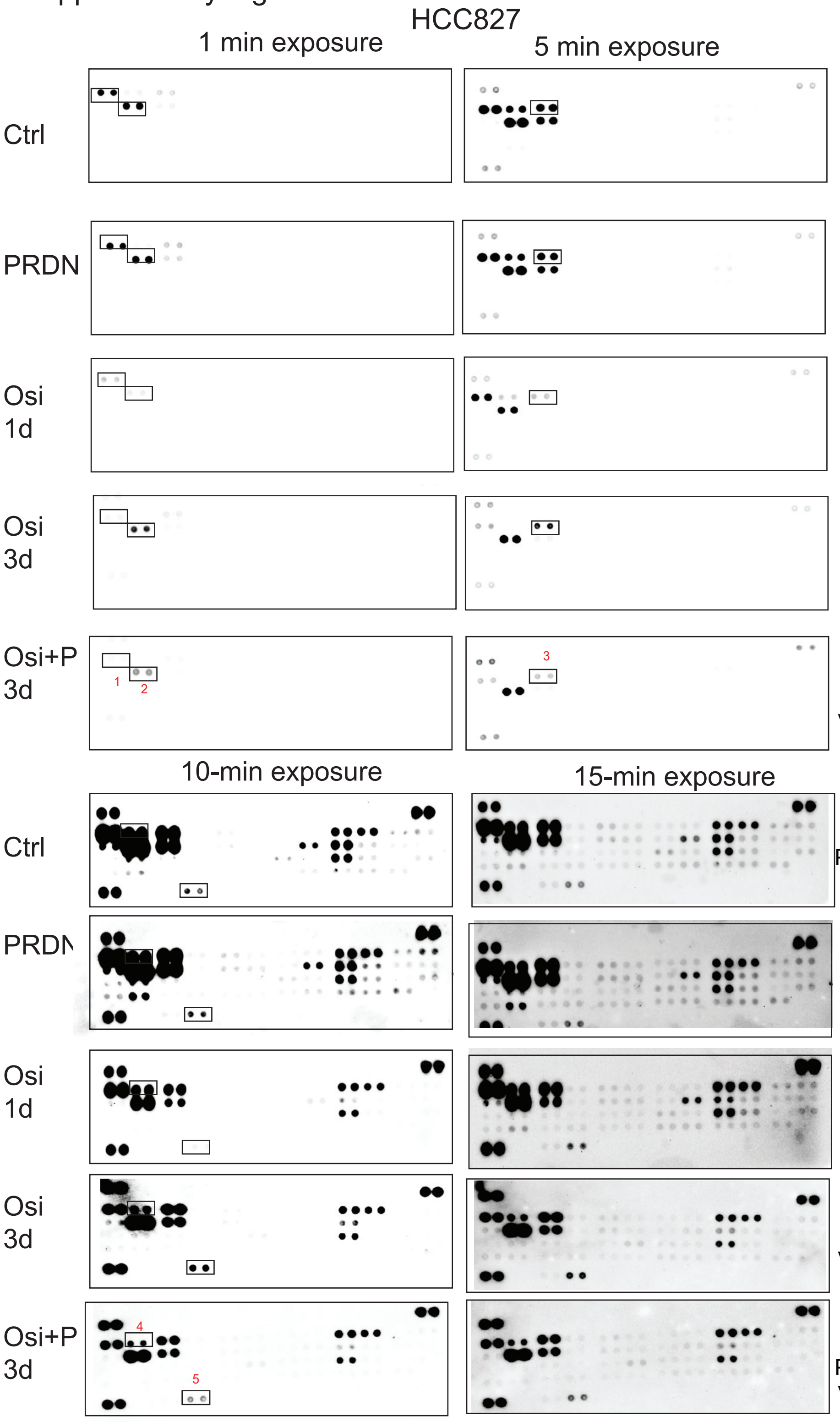

1.EGFR 2.MET 3.ERBB3 4.ERBB2 5.RYK

	Ctrl	P-3d	Osi-1d	Osi-3d	PO-3d
MET	0	-0.12	-5.35	-0.76	-4.21
EGFR	0	-0.04	-3.26	-5.75	-6.12
ERBB3	0	-0.09	-3.34	-1.04	-3.39
ERBB2	0	-0.27	-0.49	-0.07	-1.25
RET	0	0.32	-3.68	-2.24	-3.58
CSFR	0	0.47	-4.12	-9.93	-4.92
RYK	0	0.41	-4.82	0.91	-0.47
MSP	0	-0.18	-0.36	-0.35	-0.45
MER	0	0.20	-0.27	-0.72	-1.63
EPHA1	0	-0.06	0.30	-0.65	-0.24
INSR	0	0.09	0.59	-0.23	0.26
IGFR	0	0.14	0.60	-0.34	-2.03
TRKA	0	0.45	-0.48	0.35	-1.57
DTK	0	0.48	0.86	-1.01	-0.47
ROR1	0	0.15	0.22	-0.88	1.65
EPHA2	0	-0.20	0.60	-0.93	-0.56
DDR2	0	0.12	-0.58	-1.12	-0.63
AXL	0	0.08	0.49	-0.99	-0.35
EPHA5	0	-0.09	-0.17	-1.05	-0.71
EPHA4	0	0.30	1.27	-0.54	-0.65
EPHA7	0	1.43	-0.01	-0.18	-0.91
EPHA10	0	0.22	0.80	-0.61	-0.58
VEGFR3	0	-0.60	-0.71	-0.76	-0.24
ROR2	0	-0.05	0.49	-0.42	-0.37
DDR1	0	-0.40	-0.55	-0.79	-0.50
TIE1	0	0.37	1.07	-0.86	-0.38
FGFR1	0	-0.14	-0.59	0.03	-0.17
PDGFRB	0	-0.02	-0.55	0.09	-0.10
EPHA6	0	0.35	-0.95	0.55	-0.31
MUSK	0	-0.48	-0.86	-0.37	-0.05
SCFR	0	0.08	-0.34	-0.13	0.06
ALK	0	-0.59	-0.29	-0.33	-0.03
EPHA3	0	-0.31	0.79	-0.12	-0.23
EPHB3	0	-1.08	-0.94	-0.11	-0.10
TRKB	0	-0.54	-0.98	0.48	-0.04
FGFR4	0	0.14	-0.34	-0.07	0.12
FGFR2	0	-0.19	-0.36	0.11	0.27
FLT3	0	-0.18	-0.65	-0.02	0.33
EPHB6	0	-0.37	-0.78	-0.31	0.02
EPHB2	0	-0.50	-0.62	0.41	0.13
EPHB1	0	-1.37	-1.11	0.80	-0.04
FGFR3	0	-0.10	-0.41	0.02	0.37
VEGFR2	0	-0.57	-0.55	0.39	0.79
TIE2	0	-0.11	-0.96	1.27	0.31
EPHB4	0	-0.40	-0.78	0.11	0.22
ERBB4	0	-0.10	-0.21	0.55	0.31
PDGFRA	0	-1.13	-0.71	0.59	0.29
VEGFR1	0	-0.82	-0.59	0.45	0.38
TRKC	0	-1.16	-0.85	0.75	0.28

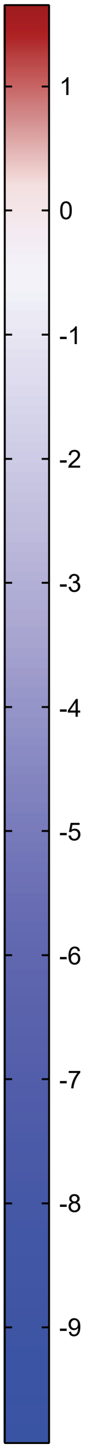

Supplementary Figure 5.

Osimertinib-induced bypass RTK signaling is suppressed by prednisolone in RTK arrays

HCC827 cells were treated with control vehicle for 3 days, 10 nM osimertinib for 1 and 3 days, osimertinib in combination with 10 μM prednisolone for 3 days. RTK Array was performed as described in the Methods.

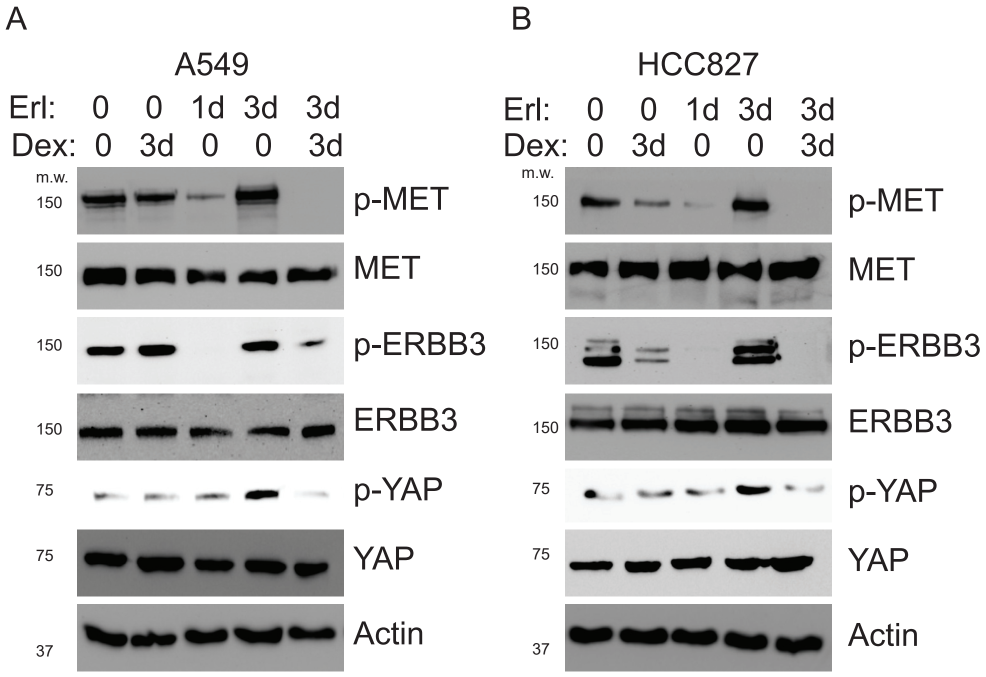
A
A549
Erl: 0 0 1d 3d 3d
Dex: 0 3d 0 0 3d
m.w.
150
p-MET
150
MET
150
p-ERBB3
150
ERBB3
75
p-YAP
75
YAP
37
Actin
B
HCC827
Erl: 0 0 1d 3d 3d
Dex: 0 3d 0 0 3d
m.w.
150
p-MET
150
MET
150
p-ERBB3
150
ERBB3
75
p-YAP
75
YAP
37
Actin

Supplementary Figure 6.

Erlotinib-induced bypass RTK signaling is suppressed by dexamethasone

A549 and HCC827 cells were treated with control vehicle for 3 days, 1 μM (A549) or 100 nM (HCC827) erlotinib for 1 and 3 days, erlotinib in combination with 10 μM dexamethasone for 3 days. Cell lysates were subjected into WB for detecting the indicated proteins. Image show one representative result from at least 3 independent experiments.

Supplementary Figure 7 Gong et al.

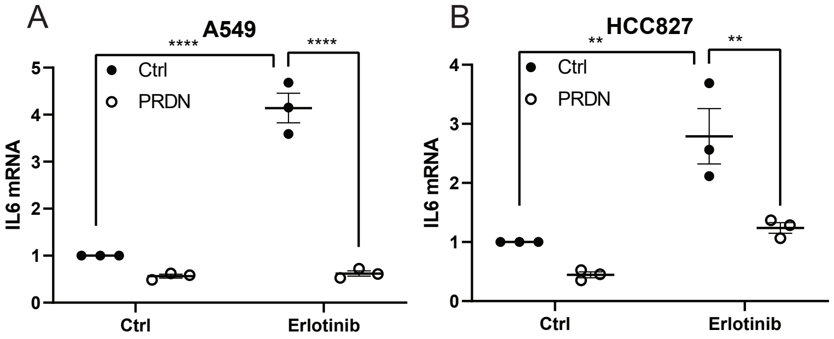

Supplementary Figure 7.

Erlotinib-induced upregulation of IL-6 is suppressed by prednisolone

A549 and HCC827 cells were treated with 1 μM (A549) or 100 nM (HCC827) erlotinib and/or 10 μM prednisolone for 1 day. Real-time PCR was performed to detect IL6 mRNA levels. 3 independent experiments were performed, showing each value and mean ± SEM. *: $p < 0.05$, **: $p < 0.01$, ***: $p < 0.001$, ****: $p<0.0001$, by two-way ANOVA, adjusted by Bonferroni's test. The statistical analysis above was performed on Graphpad Prism 9.0.0.p=2e-7,4e-5,0.005,0.003.

Supplementary Figure 8 Gong et al.

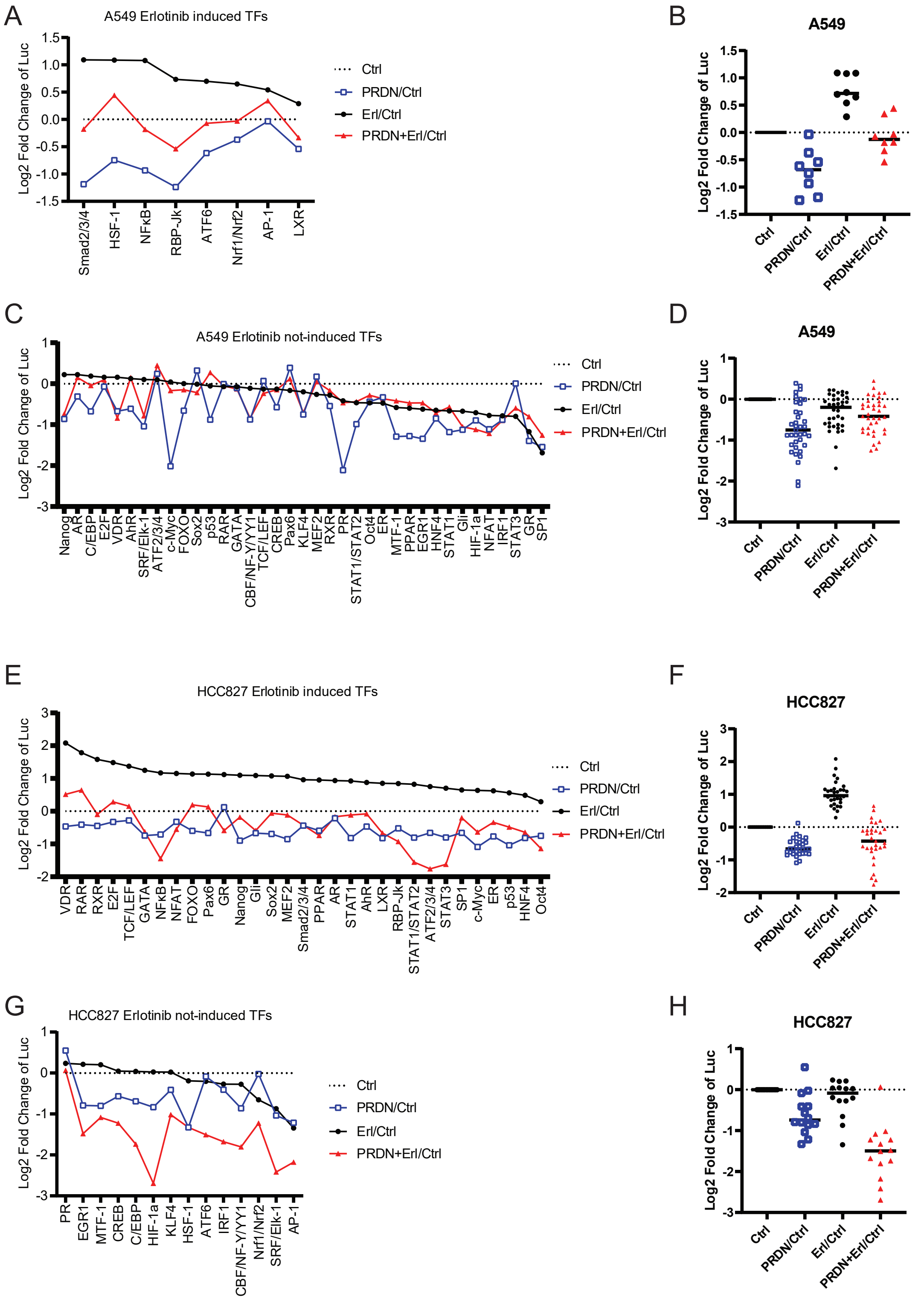

Supplementary Figure 8.

Erlotinib-induced activation of transcription factors and transcription repression by prednisolone

A-H. A549 and HCC827 cells were treated with 1 μM (A549) or 100 nM (HCC827) erlotinib and/or 10 μM prednisolone for 1 day. The activation of 45 transcription factors was examined by Cignal 45-Pathway Reporter Array (Qiagen). Each factor's luciferase activity was shown in individual and summarized dot plots. Bar means median.

Supplementary Figure 9 Gong et al.

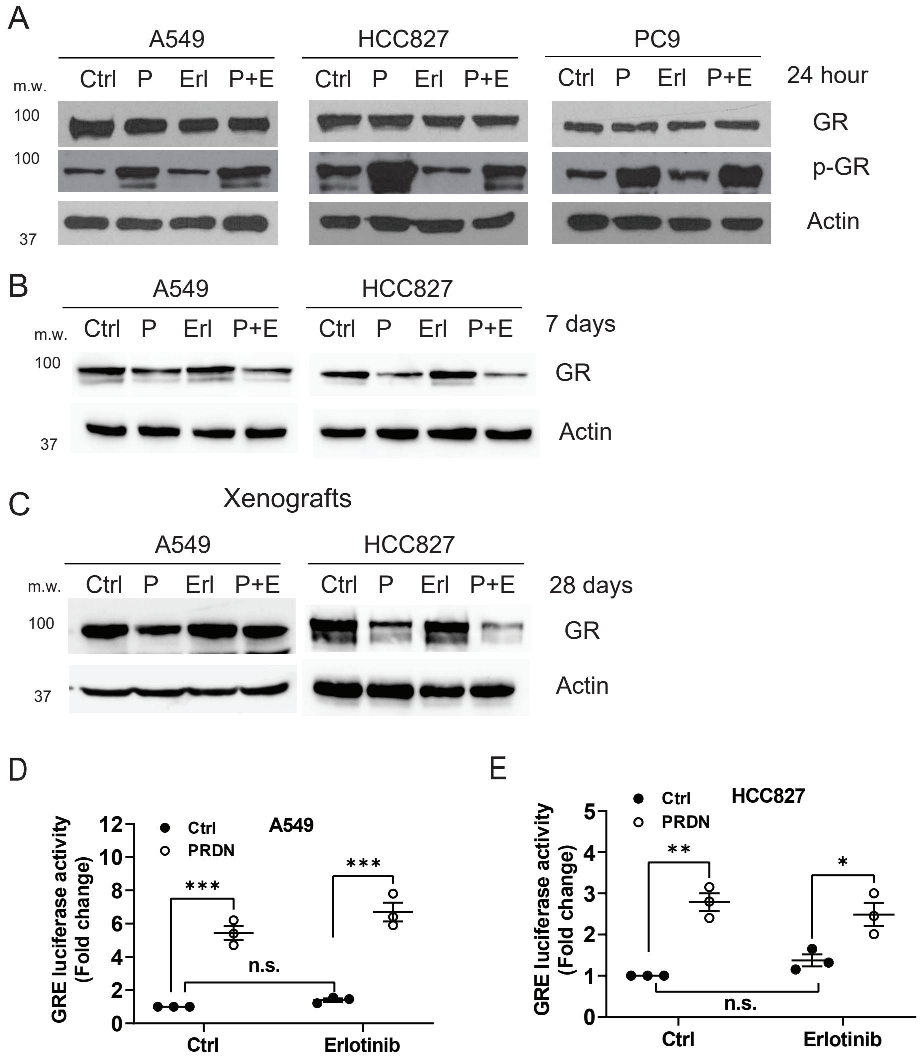

Supplementary Figure 9.

The effects of glucocorticoids and EGFR inhibition on the glucocorticoid receptor

A-B. A549, HCC827 and PC9 cells were treated with control vehicle, 10 μM prednisolone (P), 1 μM (A549) or 100 nM (HCC827 and PC9) erlotinib (Erl), erlotinib in combination with prednisolone for 24 hours or 7 days. Cell lysates were subjected into WB for detecting the indicated proteins. Images show one representative result from at least 3 independent experiments. **C.** A549 cells were s.c. injected into nude mice. Once tumors formed, mice were divided into following four groups (n=3 per group), control vehicle, erlotinib 100 mg/kg/day, prednisone 5 mg/kg/day (PRDN), combination of erlotinib and PRDN. Four weeks later, tumors were collected, protein was extracted, NR3C1 levels were determined by WB. WB images show one representative result. Similar experiments were performed on HCC827, except that erlotinib was used at a dose of 12.5 mg/kg/day. **D-E.** A549 or HCC827 cells were transiently transfected with GRE 7.3 luciferase reporter or empty vector and treated with control vehicle, 10 μM prednisolone, 1 μM (A549) or 100 nM (HCC827) erlotinib, erlotinib in combination with prednisolone for 24 hours. Luciferase activities are expressed as fold change versus control. Experiments were performed in 3 independent times, showing each value and mean ± SEM. ***: $p < 0.001$, **: $p<0.01$, *: $p<0.05$, n.s: not significant. p=3e-5,2e-6,0.006,0.03

Supplementary Figure 10 Gong et al.

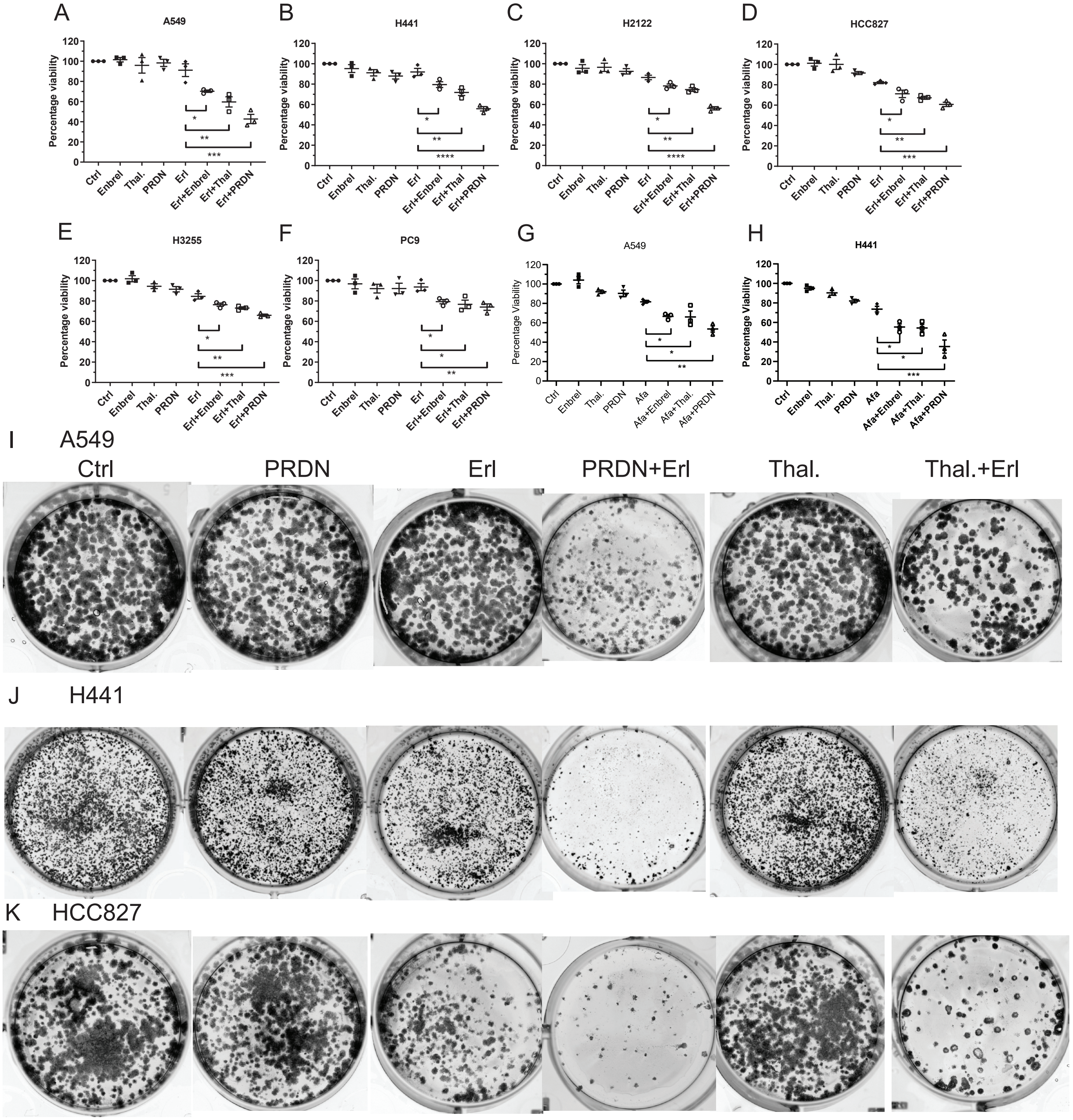

Supplementary Figure 10.

Effect of combination treatments on cell survival

A-C. EGFR wt cell lines were treated with 1 μM erlotinib with or without 100 μg/mL etanercept, 10 μM thalidomide, 10 μM prednisolone for 72 hours. AlamarBlue assay was used for determining cell viability. **D-F.** Similar experiments were performed on EGFR mutant lines, and erlotinib was used at 100 nM for HCC827, and 10 nM for H3255 and PC9. **G-H.** Similar experiments using 1 μM afatinib on EGFR wt lines. 3 independent experiments were performed, showing each value and mean ± SEM. *: $p < 0.05$, **: $p < 0.01$, ***: $p < 0.001$, ****: $p<0.0001$, by two-way ANOVA, adjusted by Bonferroni's test. The statistical analysis above was performed on Graphpad Prism 9.0.0**.** **I-J.** NSCLC cell lines were planted in 6-well plates and treated with 1 μM erlotinib, 10 μM thalidomide, and 10 μM prednisolone as indicated for 14 days. Cell colonies were fixed by 100% methanol and then stained by 0.5% crystal violet in 25% methanol. Images captured by a scanner are representative of at least 3 independent experiments. p=0.03,0.006,0.001; 0.04,0.007, 2e-4;0.03.0.004,5e-5;0.02,0.01,0.0005;0.04,0.008,0.001;0.03.0.02,0.003;0.02,0.04,0.008;0.02,0.02,3e-4(A-H).

Supplementary Figure 11 Gong et al.

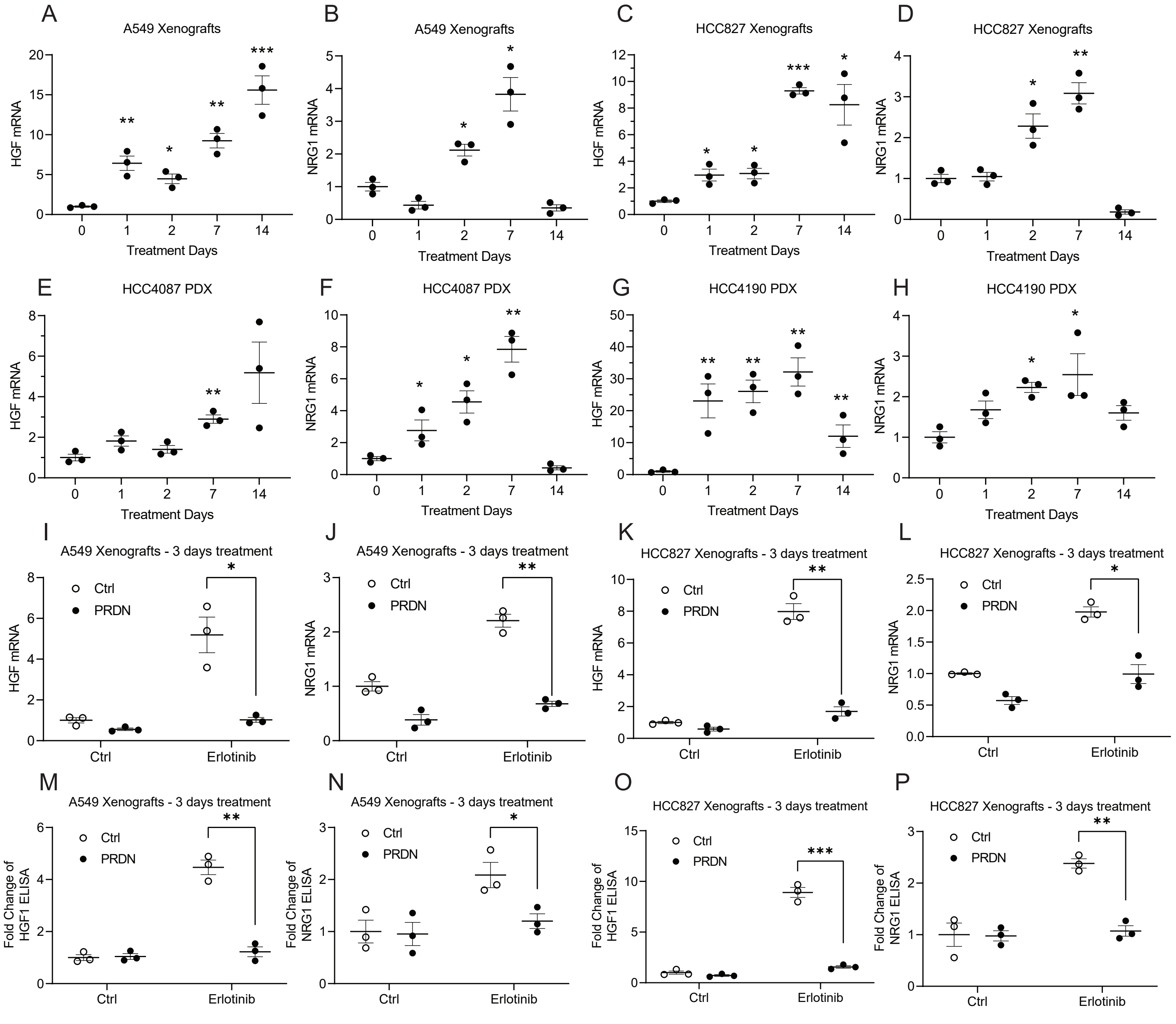

Supplementary Figure 11.

Upregulation of RTK ligands by EGFR inhibition in mouse tumors

A-D. 1 million A549 cells or 2 million HCC827 cells were s.c. injected into 15 nude mice each line. Once tumors formed, mice were divided into 5 groups (n=3) and received 100 mg/kg/day (A549) or 12.5 mg/kg/day (HCC827) erlotinib for 0, 1, 2, 4, 7, 14 days. Tumors were extracted from sacrificed mice. HGF and NRG1 mRNA were determined by real-time PCR. **E-H.** Similar experiments were performed on EGFR wt (HCC4087) and mutant (HCC4190) PDX. **I-J.** 12 nude mice were s.c. injected with 1 million A549 cells. After tumor formation, mice were divided into 4 groups (n=3) and received 100 mg/kg/day erlotinib and/or 5 mg /kg prednisone for 3 days. HGF and NRG1 mRNA levels were tested from the extracted tumors. **K-L.** A similar experiment in HCC827 tumors, using 12.5 mg/kg/day erlotinib. **M-P.** HGF and NRG1 protein levels were also examined in these 3-day treated tumors. Data represent each value and mean ± SEM. *: $p < 0.05$, **: $p < 0.01$, ***: $p < 0.001$, ****: $p<0.0001$, by one-way ANOVA adjusted by Dunnett's test (A-H), and two-way ANOVA adjusted by Bonferroni's test (I-P). The statistical analysis above was performed on Graphpad Prism 9.0.0. p = 0.005, 0.03, 0.003,0.001;0.01,0.03;0.03,0.02,2e-7,0.05;0.03,0.006; 0.003;0.04,0.03,0.01;0.009,0.008,0.009,0.005;0.02,0.05(A-H), and 0.03,0.005.0.008,0.04,0.006,0.035,1e-4,0.004(I-P).

Supplementary Figure 12

Gong et al.

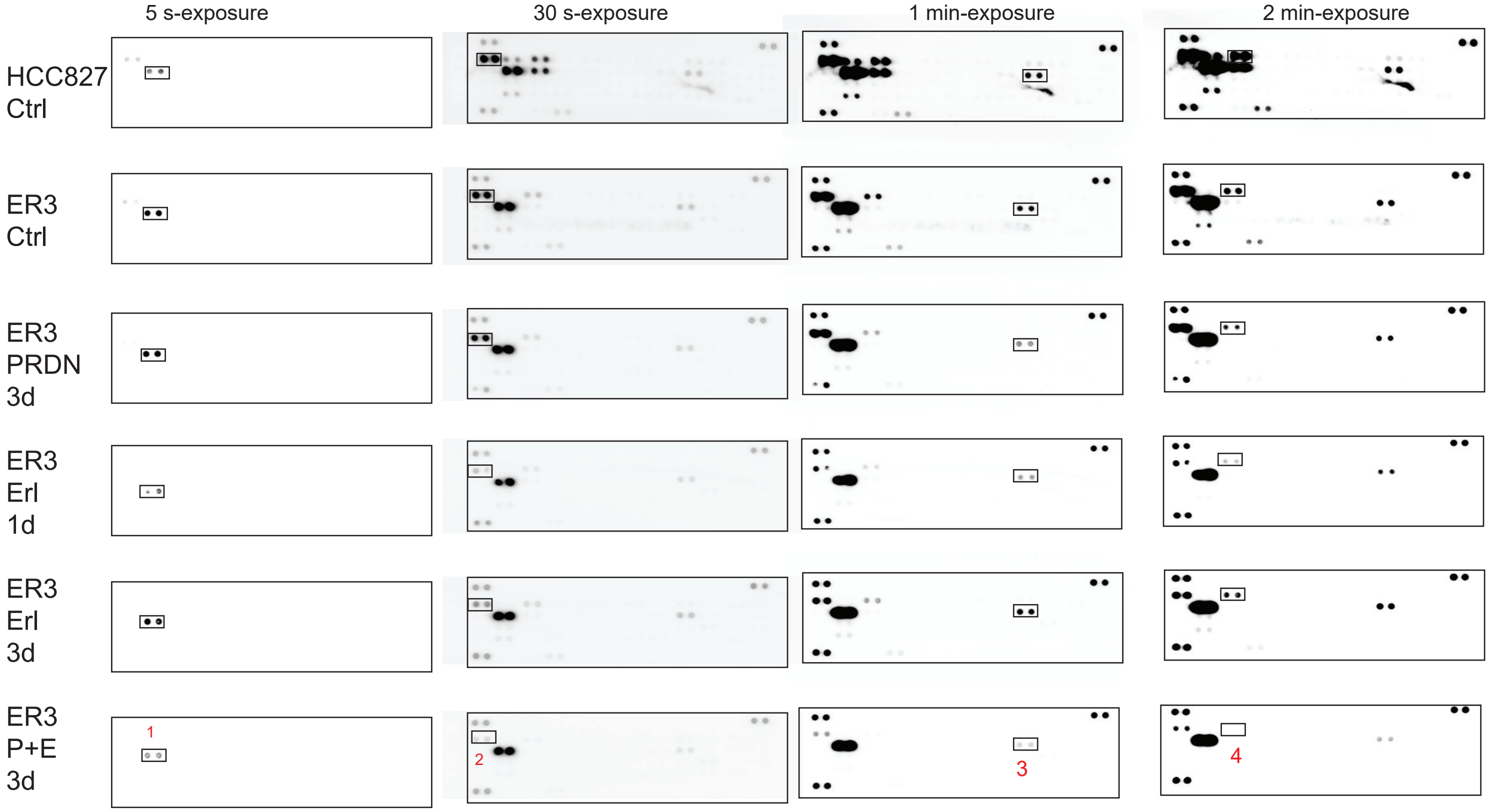

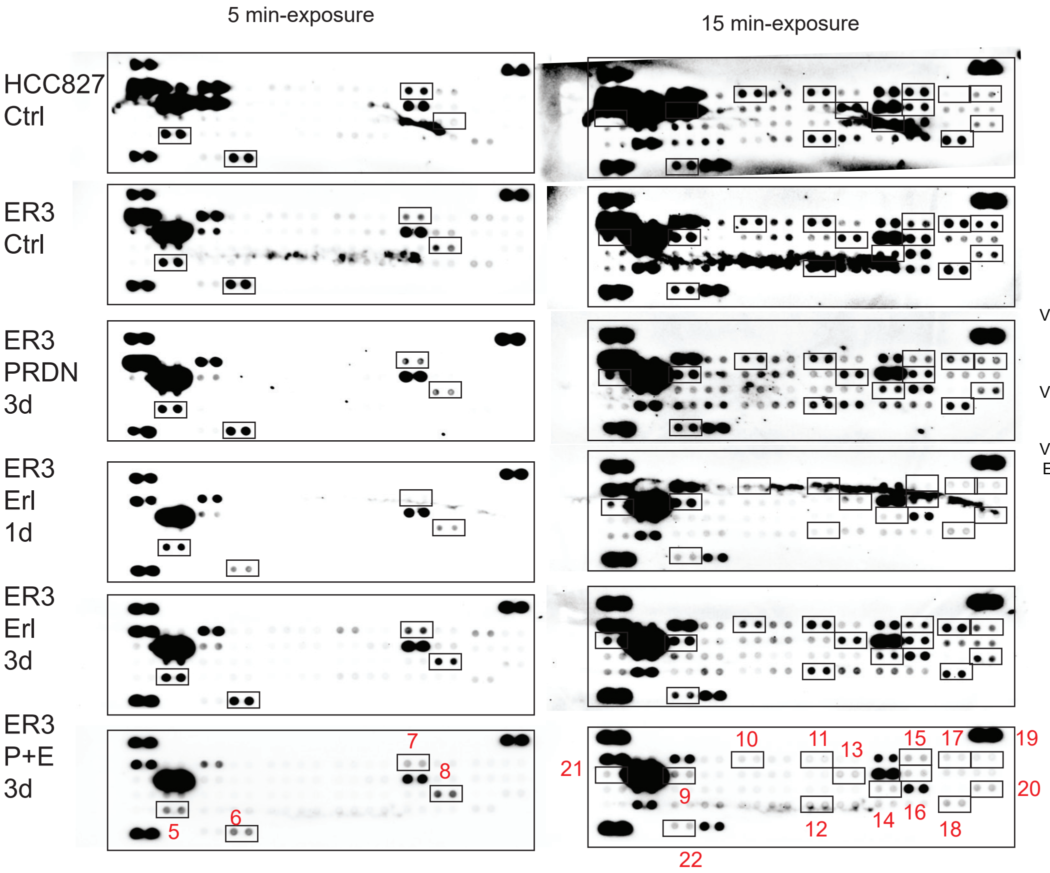

1.MET 2.EGFR 3.RET 4. ERBB3 5.EPHA7 6. RYK 7.INSR 8.EPHA2 9. MSP 10. FGFR1 11. FGFR3 12. ALK 13. CSFR 14.EPHA1 15.IGFR 16.ROR1 17. AXL 18. EPHA10 19. DTK 20.EPHA4 21. MER 22. EPHB3

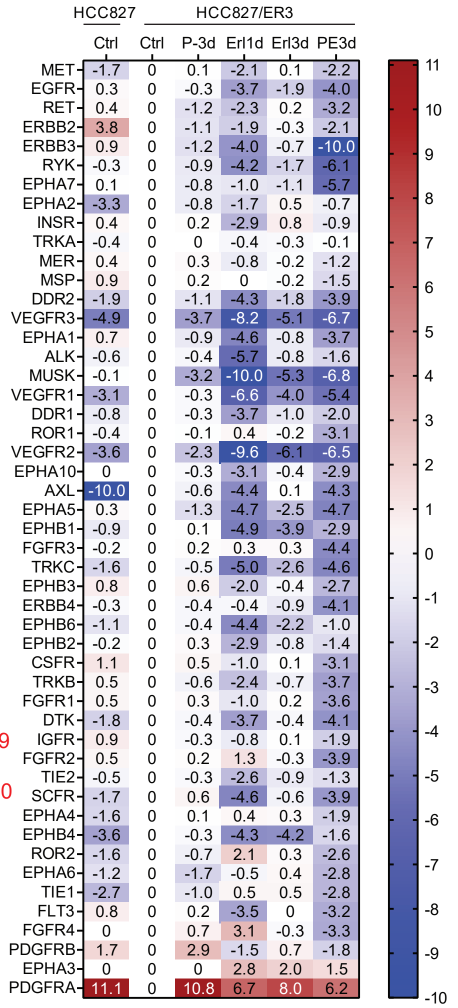

	HCC827	HCC827/ER3				
	Ctrl	Ctrl	P-3d	Erl1d	Erl3d	PE3d
MET	-1.7	0	0.1	-2.1	0.1	-2.2
EGFR	0.3	0	-0.3	-3.7	-1.9	-4.0
RET	0.4	0	-1.2	-2.3	0.2	-3.2
ERBB2	3.8	0	-1.1	-1.9	-0.3	-2.1
ERBB3	0.9	0	-1.2	-4.0	-0.7	-10.0
RYK	-0.3	0	-0.9	-4.2	-1.7	-6.1
EPHA7	0.1	0	-0.8	-1.0	-1.1	-5.7
EPHA2	-3.3	0	-0.8	-1.7	0.5	-0.7
INSR	0.4	0	0.2	-2.9	0.8	-0.9
TRKA	-0.4	0	0	-0.4	-0.3	-0.1
MER	0.4	0	0.3	-0.8	-0.2	-1.2
MSP	0.9	0	0.2	0	-0.2	-1.5
DDR2	-1.9	0	-1.1	-4.3	-1.8	-3.9
VEGFR3	-4.9	0	-3.7	-8.2	-5.1	-6.7
EPHA1	0.7	0	-0.9	-4.6	-0.8	-3.7
ALK	-0.6	0	-0.4	-5.7	-0.8	-1.6
MUSK	-0.1	0	-3.2	-10.0	-5.3	-6.8
VEGFR1	-3.1	0	-0.3	-6.6	-4.0	-5.4
DDR1	-0.8	0	-0.3	-3.7	-1.0	-2.0
ROR1	-0.4	0	-0.1	0.4	-0.2	-3.1
VEGFR2	-3.6	0	-2.3	-9.6	-6.1	-6.5
EPHA10	0	0	-0.3	-3.1	-0.4	-2.9
AXL	-10.0	0	-0.6	-4.4	0.1	-4.3
EPHA5	0.3	0	-1.3	-4.7	-2.5	-4.7
EPHB1	-0.9	0	0.1	-4.9	-3.9	-2.9
FGFR3	-0.2	0	0.2	0.3	0.3	-4.4
TRKC	-1.6	0	-0.5	-5.0	-2.6	-4.6
EPHB3	0.8	0	0.6	-2.0	-0.4	-2.7
ERBB4	-0.3	0	-0.4	-0.4	-0.9	-4.1
EPHB6	-1.1	0	-0.4	-4.4	-2.2	-1.0
EPHB2	-0.2	0	0.3	-2.9	-0.8	-1.4
CSFR	1.1	0	0.5	-1.0	0.1	-3.1
TRKB	0.5	0	-0.6	-2.4	-0.7	-3.7
FGFR1	0.5	0	0.3	-1.0	0.2	-3.6
DTK	-1.8	0	-0.4	-3.7	-0.4	-4.1
IGFR	0.9	0	-0.3	-0.8	0.1	-1.9
FGFR2	0.5	0	0.2	1.3	-0.3	-3.9
TIE2	-0.5	0	-0.3	-2.6	-0.9	-1.3
SCFR	-1.7	0	0.6	-4.6	-0.6	-3.9
EPHA4	-1.6	0	0.1	0.4	0.3	-1.9
EPHB4	-3.6	0	-0.3	-4.3	-4.2	-1.6
ROR2	-1.6	0	-0.7	2.1	0.3	-2.6
EPHA6	-1.2	0	-1.7	-0.5	0.4	-2.8
TIE1	-2.7	0	-1.0	0.5	0.5	-2.8
FLT3	0.8	0	0.2	-3.5	0	-3.2
FGFR4	0	0	0.7	3.1	-0.3	-3.3
PDGFRB	1.7	0	2.9	-1.5	0.7	-1.8
EPHA3	0	0	0	2.8	2.0	1.5
PDGFRA	11.1	0	10.8	6.7	8.0	6.2

Supplementary Figure 12.

Bypass RTK activation is NSCLC cell lines rendered secondarily resistant to erlotinib and suppression by prednisolone

HCC827 and its derived erlotinib-resistant ER3 lines were treated control vehicle for 3 days, 10 μM prednisolone for 3 days, 1 μM erlotinib for 1 day and 3 days, and prednisolone + erlotinib for 3 days. Proteome Profiler Human Phospho-RTK Array (R&D, ARY001B) profiling was performed. Images were captured by ChemiDoc MP Imaging System (Bio-Rad) at exposures with varying time, processed by Image Lab 6.0.1 (Bio-Rad), and quantitated by HLImage++ (Western Vision Software). Some representative RTKs were framed up and footnoted. The quantitation was based on the longest exposure, except for the framed RTKs in shorter exposures to avoid overexposure. RTKs were ranked by basal expression of ER3. The log 2-fold changes of every treatment condition compared to ER3 were show in the heatmap.

Supplementary Figure 13

Gong et al.

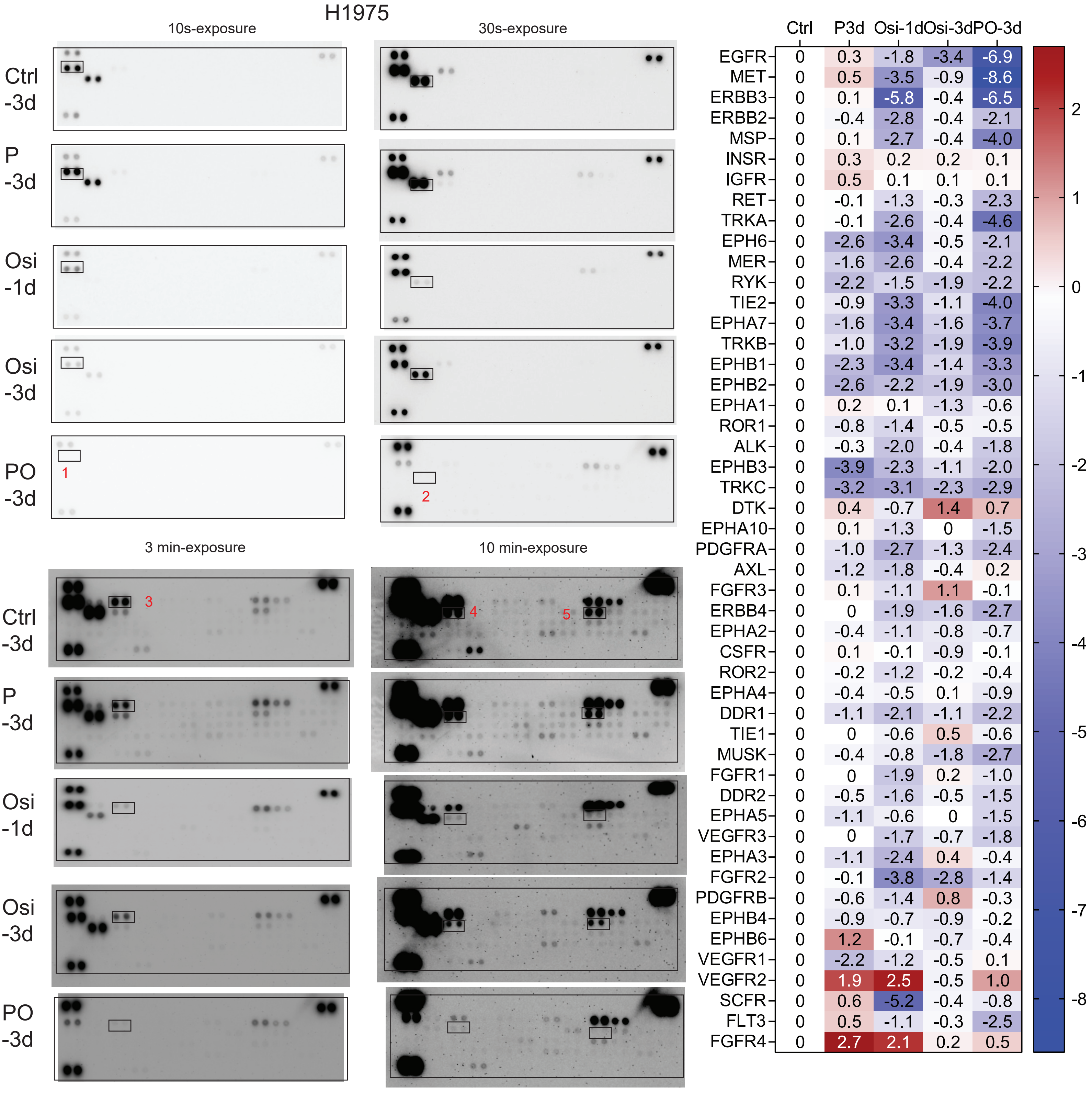

1. EGFR 2. MET 3. ERBB3 4.MSP 5. RET

Supplementary Figure 13.

Bypass RTK activation in H1975 cells and suppression by prednisolone

A similar RTK array experiment was performed on EGFR L858R and T790M mutation line H1975. Osimertinib at 10 nM was used instead of erlotinib.

Supplementary Figure 14

Gong et al.

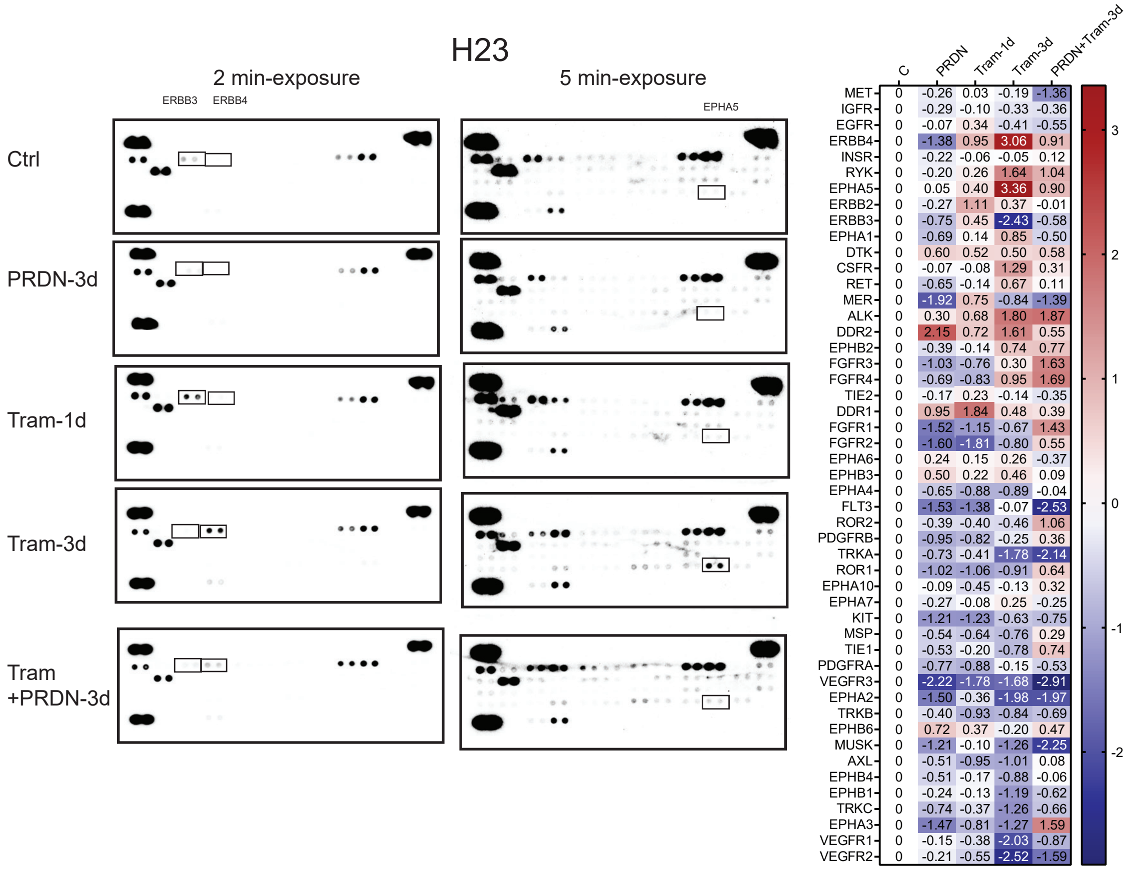

Supplementary Figure 14.

Bypass RTK activation in H23 cells and suppression by prednisolone

A similar RTK array experiment was performed on KRAS and ATM mutant H23 lung cancer line, which is sensitive to a MEK inhibitor, trametinib at a dose of 10 nM.

Supplementary Figure 15

Gong et al.

H1703

10-min exposure

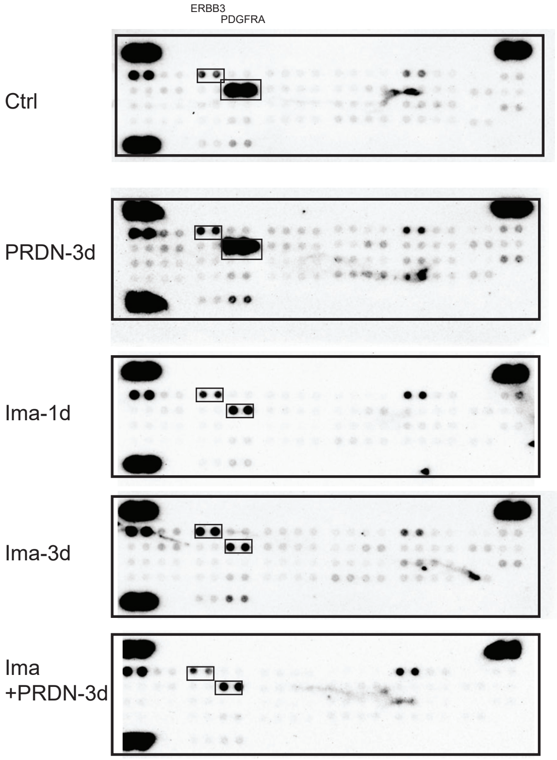

	Ctrl	PRDN	Ima-1d	Ima-3d	PI-3d
PDGFRA	0	-0.06	-1.14	-1.47	-1.24
EGFR	0	-0.38	-0.40	-0.02	-0.31
RET	0	2.29	-0.99	-0.83	-1.28
ERBB3	0	-0.85	-0.47	0.36	-0.85
INSR	0	-0.80	0.23	-0.46	0.36
RYK	0	-1.62	-2.55	-0.63	-2.86
CSFR	0	-0.35	-1.24	-0.70	-0.83
MSP	0	-0.76	-3.60	-2.34	-2.78
EPHA4	0	-1.14	-2.79	-0.70	-3.87
MUSK	0	1.87	-0.11	-0.56	1.55
EPHA10	0	-0.95	-1.85	0.77	0.87
DTK	0	-1.43	0.95	-0.91	-2.57
EPHA2	0	0.26	-1.22	1.04	-2.10
TRKC	0	-2.56	-7.13	-6.07	-4.80
MER	0	-1.49	-2.09	-1.27	-2.38
ERBB4	0	-1.57	-2.83	-0.44	-2.71
ROR2	0	-1.22	-1.52	-0.46	-2.14
MET	0	-2.36	-2.74	-0.43	-2.66
ERBB2	0	-2.88	-2.29	-0.41	-1.99
TIE2	0	-1.98	-2.53	-1.91	-2.08
EPHB3	0	-1.20	-2.13	-0.27	-1.43
EPHB2	0	-1.88	-2.00	-0.77	-1.36
DDR1	0	-3.03	-3.43	-2.05	-3.17
DDR2	0	-4.49	-5.19	-3.40	-4.71
ALK	0	-2.27	-1.80	-0.39	-2.21
EPHA10	0	-1.42	-1.85	2.40	-1.99
TIE1	0	-1.37	-0.99	-0.66	-2.20
VEGFR3	0	-2.43	-2.89	-2.14	-0.58
ROR1	0	-1.23	-1.77	-0.76	-1.51
IGFR	0	-1.76	-1.19	-1.21	-0.73
TRKB	0	-1.78	-2.64	-2.23	-1.51
PDGFB	0	-2.61	-2.37	-0.59	-2.13
FGFR1	0	-2.74	-2.45	-0.77	-2.19
EPHA6	0	-2.39	-2.28	-1.25	-1.64
EPHA5	0	-2.81	-1.84	-0.97	-2.46
VEGFR2	0	-3.01	-4.10	-2.91	-2.47
VEGFR1	0	-2.11	-2.81	-1.42	-0.95
EPHB1	0	-2.56	-2.24	-1.19	-0.59
EPHA3	0	-0.56	-0.53	-0.02	-1.17
AXL	0	-2.96	-3.28	-1.06	-1.44
EPHB4	0	-2.03	-1.13	-0.38	0.07
TRKA	0	-5.33	-2.71	-2.13	-2.28
FGFR4	0	-4.14	-2.24	0.24	-1.67
EPHA7	0	-4.87	-2.76	-1.87	-1.66
FGFR3	0	-5.02	-2.60	-1.41	-2.21
FGFR2	0	-6.11	-2.65	-0.99	-2.13
FLT3	0	-5.00	-1.20	-0.65	1.31
EPHB6	0	-5.79	-1.79	-0.90	-1.68
SCFR	0	-7.79	-2.74	-0.69	-0.01

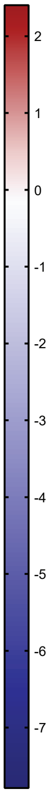

Supplementary Figure 15.

Bypass RTK activation in H1703 cells and suppression by prednisolone

A similar RTK array experiment was performed on PDGFR amplification H1703 lung cancer line, which is moderately sensitive/resistant to a BCR-ABL/PDGFR dual inhibitor, imatinib at a dose of 100 nM.

Supplementary Figure 16

Gong et al.

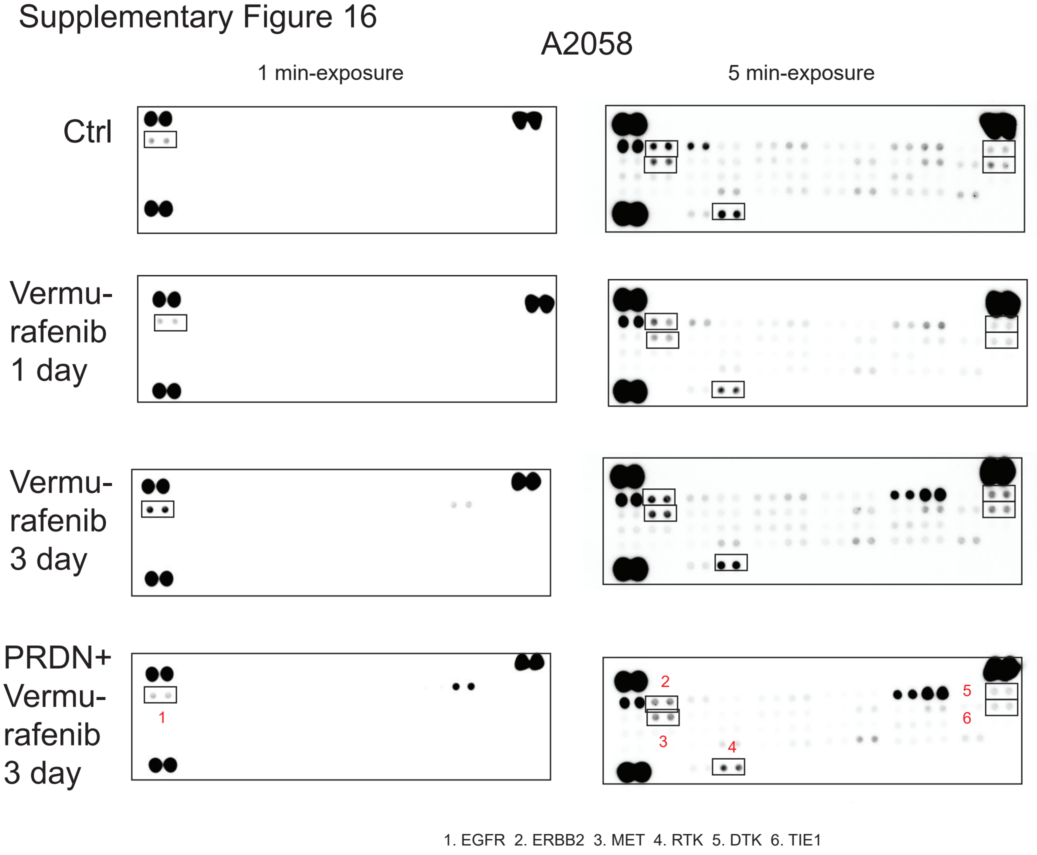

	Ctrl	Verm - 1d	Verm - 3d	P+V - 3d
EGFR	0	-2.07	1.72	-0.66
RYK	0	-0.93	0.08	-0.95
ERBB2	0	-1.11	-0.03	-1.06
ERBB3	0	-1.93	-2.60	-3.85
MET	0	-1.25	0.42	-0.46
IGFR	0	0.01	2.72	2.81
TIE1	0	-1.58	0.36	-1.41
ROR1	0	-2.16	0.40	-1.06
EPHB2	0	-2.07	-0.38	-1.65
EPHA10	0	-2.47	-0.12	-1.53
FGFR2	0	-1.67	-0.19	-1.99
DDR1	0	-1.31	0.27	0.15
INSR	0	-0.69	2.62	2.75
DTK	0	-0.14	1.64	-0.44
MER	0	-1.48	-0.31	-2.13
EPHB3	0	-1.88	-0.52	-1.89
EPHA1	0	-2.33	-0.12	-1.74
CSFR	0	-1.66	0.58	-1.18
TIE2	0	-1.66	-0.46	-1.97
ROR2	0	-2.30	-0.26	-1.86
DDR2	0	-2.71	-0.27	-1.94
EPHB6	0	-2.28	-0.20	-1.52
FGFR1	0	-1.56	0	-2.10
EPHA5	0	-2.31	-0.12	-1.95
TRKA	0	-1.71	-0.01	-1.79
PDGFRB	0	-1.81	0.04	-1.97
EPHA6	0	-1.78	-0.50	-1.86
PDGFRA	0	-2.67	-0.53	-2.84
TRKC	0	-1.90	-0.29	-2.01
ERBB4	0	-1.56	0.10	-2.43
ALK	0	-2.42	-0.04	-1.44
VEGFR2	0	-1.23	0.71	-1.59
RET	0	-2.45	-0.09	-1.44
SCFR	0	-1.48	0.30	-1.21
MUSK	0	-1.78	-0.14	-1.77
MSP	0	-2.12	0.16	-1.82
FGFR3	0	-1.43	-0.07	-1.83
EPHA4	0	-2.31	-0.30	-2.21
AXL	0	-2.06	0.18	-1.77
FGFR4	0	-1.37	0.47	-1.43
EPHA2	0	-3.33	1.85	0.14
VEGFR1	0	-2.36	1.36	-1.69
TRKB	0	-2.09	-0.30	-2.20
VEGFR3	0	-2.42	-0.22	-1.90
EPHA7	0	-2.38	-0.20	-2.44
EPHB4	0	-2.34	-0.20	-1.82
EPHB1	0	-1.77	0.35	-1.96
EPHA3	0	-4.39	-0.18	-3.58
FLT3	0	-2.55	-0.40	-2.68

Color scale: 2, 1, 0, -1, -2, -3, -4

1

Supplementary Figure 16.

Bypass RTK activation in A2058 cells and suppression by prednisolone

A similar RTK array experiment was performed on A2058 melanoma cell line, which is BRAF V600E mutant but resistant to a BRAF V600E inhibitor, vemurafenib at a dose of 1 μM.

Supplementary Figure 17

Gong et al.

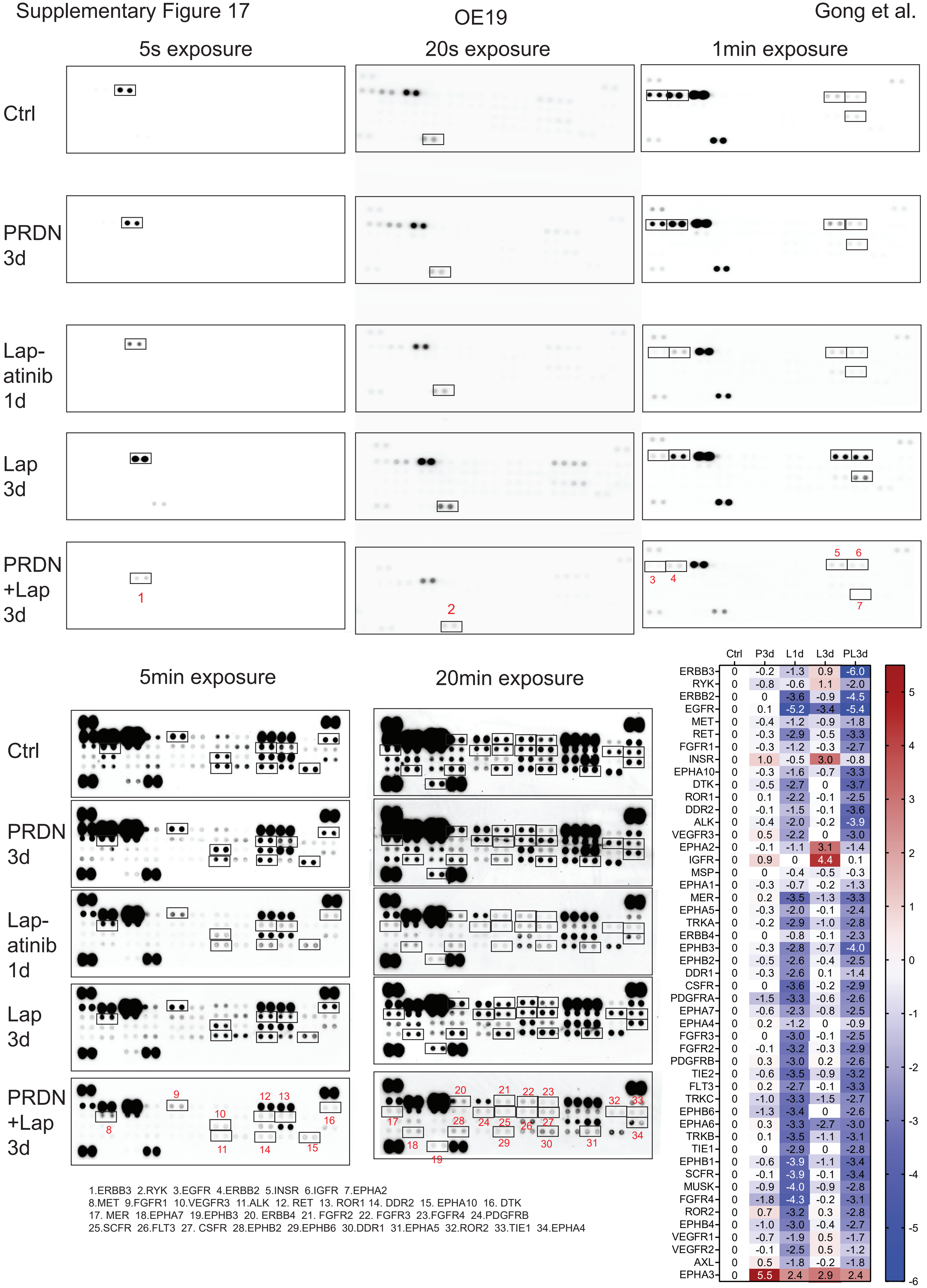

Supplementary Figure 17.

Bypass RTK activation in OE19 cells and suppression by prednisolone

A similar RTK array experiment was performed on an ERBB2 amplification esophageal adenocarcinoma line OE19, which is sensitive to an ERBB2 inhibitor, lapatinib at a dose of 10 nM.

Supplementary Figure 18 Gong et al.

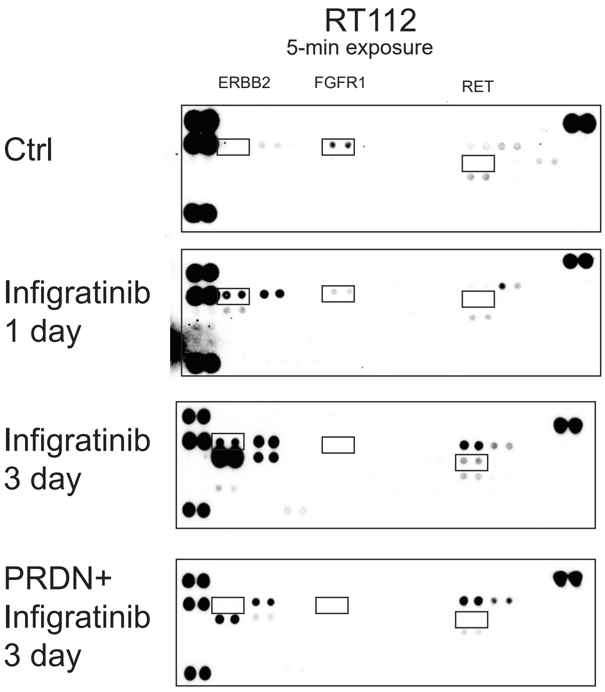

	Ctrl	Infi-1d	Infi -3d	PRDN+Infi -3d
EGFR	0	-0.19	-0.59	-0.33
FGFR1	0	-2.58	-6.52	-8.17
MER	0	0.69	1.30	-3.00
EPHA1	0	-0.92	-0.22	-1.67
IGFR	0	1.16	0.95	1.50
EPHA6	0	3.47	-3.07	-5.26
ROR2	0	-5.24	-5.17	-5.92
ERBB3	0	4.03	4.94	3.68
INSR	0	-1.57	4.71	4.70
FGFR2	0	-3.03	-3.03	-3.05
EPHA2	0	-0.71	1.30	-1.78
ROR1	0	0.03	-0.33	-1.61
PDGFRB	0	-1.46	-0.43	-1.46
ERBB2	0	7.93	6.89	1.48
TIE1	0	-1.01	-0.64	-1.01
ERBB4	0	-0.98	0.22	-0.98
TIE2	0	8.20	1.60	-0.94
DTK	0	0.36	2.15	0.04
VEGFR2	0	-0.53	0.63	-0.53
VEGFR3	0	-0.25	0.77	-0.44
AXL	0	-0.32	-0.36	-0.01
RYK	0	2.07	5.30	2.60
MET	0	6.84	10.57	8.48
VEGFR1	0	-0.17	-0.22	-0.21
EPHB6	0	1.39	0.65	-0.15
MUSK	0	-0.11	0.86	-0.11
CSFR	0	1.63	2.59	-0.04
EPHA10	0	0.48	-0.04	-0.04
EPHB3	0	2.71	1.39	-0.03
MSP	0	0.42	10.11	5.76
EPHB2	0	1.66	1.30	0.05
TRKB	0	0.45	2.39	0.06
RET	0	0.08	7.74	0.71
FGFR3	0	1.06	0.11	0.08
FGFR4	0	0.08	0.08	0.08
PDGFRA	0	0.08	1.06	0.08
SCFR	0	0.08	0.25	0.08
FLT3	0	0.08	0.08	0.52
TRKA	0	1.58	7.50	0.08
TRKC	0	0.08	0.12	0.19
EPHA3	0	0.08	0.08	0.08
EPHA4	0	0.08	0.08	0.08
EPHA7	0	2.92	6.54	0.08
EPHB1	0	0.08	1.02	0.08
EPHB4	0	0.15	0.12	0.08
ALK	0	0.35	1.49	0.08
DDR1	0	0.39	1.63	0.08
DDR2	0	0.08	0.64	0.08
EPHA5	0	0.08	0.87	0.08

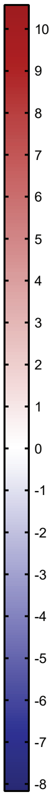

Supplementary Figure 18.

Bypass RTK activation in RT112 cells and suppression by prednisolone

A similar RTK array experiment was performed on FGFR mutant RT112 bladder cancer cell line, which is sensitive to an FGFR1-3 inhibitor, infigratinib at a dose of 10 nM.

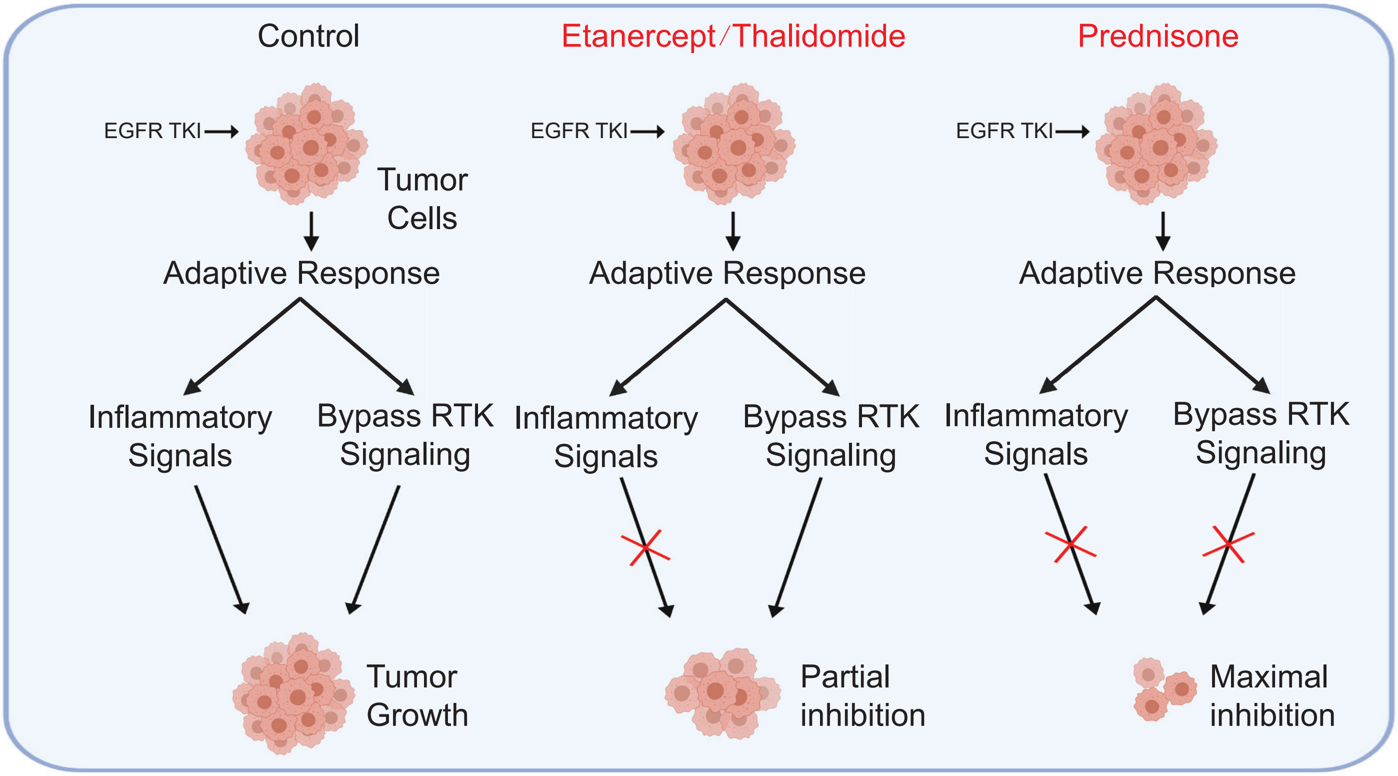
Control
Etanercept/Thalidomide
Prednisone
EGFR TKI
Tumor Cells
Adaptive Response
Inflammatory Signals
Bypass RTK Signaling
Tumor Growth
Partial inhibition
Maximal inhibition

Supplementary Figure 19.

A schematic diagram shows that prednisone suppresses the EGFR inhibition-induced adaptive response more effectively compared to etanercept or thalidomide.

EGFR inhibition results in activation of inflammatory pathways and bypass RTK signaling. Both arms of the adaptive response promote therapeutic resistance. In control vehicle treated cells, both arms of the adaptive response are active and the tumor is resistant to EGFR TKIs. The inflammatory arm of the adaptive response is blocked by etanercept, thalidomide, or prednisone. Thus, etanercept and thalidomide treatment results in a partial response to EGFR inhibition. However, only prednisone blocks the bypass RTK activation and thus is a more effective suppressor of the adaptive response to EGFR inhibition and results in a more effective suppression of tumor growth. Created with BioRender.com.
