# Supplementary material for: Comprehensive targeting of resistance to inhibition of RTK signaling pathways by using glucocorticoids
Source: Nat Commun. 2021 Dec 1;12:7014. doi: 10.1038/s41467-021-27276-7 (PMC8636603; doi:10.1038/s41467-021-27276-7)
Supplement: Supplementary file 3 — Reporting Summary [file 41467_2021_27276_MOESM3_ESM.pdf]

nature portfolio

Corresponding author(s): Amyn A. Habib

Last updated by author(s): Oct 27, 2021

Reporting Summary

Nature Portfolio wishes to improve the reproducibility of the work that we publish. This form provides structure for consistency and transparency in reporting. For further information on Nature Portfolio policies, see our Editorial Policies and the Editorial Policy Checklist.

Statistics

For all statistical analyses, confirm that the following items are present in the figure legend, table legend, main text, or Methods section.

n/a	Confirmed	
☐	☒	The exact sample size (*n*) for each experimental group/condition, given as a discrete number and unit of measurement
☐	☒	A statement on whether measurements were taken from distinct samples or whether the same sample was measured repeatedly
☐	☒	The statistical test(s) used AND whether they are one- or two-sided<br>*Only common tests should be described solely by name; describe more complex techniques in the Methods section.*
☐	☒	A description of all covariates tested
☐	☒	A description of any assumptions or corrections, such as tests of normality and adjustment for multiple comparisons
☐	☒	A full description of the statistical parameters including central tendency (e.g. means) or other basic estimates (e.g. regression coefficient) AND variation (e.g. standard deviation) or associated estimates of uncertainty (e.g. confidence intervals)
☐	☒	For null hypothesis testing, the test statistic (e.g. *F*, *t*, *r*) with confidence intervals, effect sizes, degrees of freedom and *P* value noted<br>*Give P values as exact values whenever suitable.*
☒	☐	For Bayesian analysis, information on the choice of priors and Markov chain Monte Carlo settings
☒	☐	For hierarchical and complex designs, identification of the appropriate level for tests and full reporting of outcomes
☒	☐	Estimates of effect sizes (e.g. Cohen's *d*, Pearson's *r*), indicating how they were calculated

Our web collection on statistics for biologists contains articles on many of the points above.

Software and code

Policy information about availability of computer code

Data collection	Cell viability assay, Luciferase assays, Protein concentration assay, and ELISA assay: software 1.0 installed in POLARstar Omega Microplate Reader (BMG LABTECH)<br>Real-time PCR: software installed in ViiA 7 Real-Time PCR System (Applied Biosystems).<br>RNAseq: software installed in the BGISEQ-500 system (PE150).<br>MRI imaging: software installed in a 7T small animal MRI scanner (Bruker, Rheinstetten, Germany)<br>WB and RTK array: ChemiDoc MP Imaging System (Bio-Rad)
Data analysis	Real-time PCR: Software version 1.0 installed in ViiA 7 Real-Time PCR System (Applied Biosystems) and GraphPad Prism 9.0.0.<br>RNAseq: Fastq1.4.0; STAR2.7; EdgeR3.36; Gene Set Enrichment Analysis (GSEA4.1) and ssGSEA2.0 at http://software.broadinstitute.org/gsea/index.jsp.<br>MRI, WB images: ImageJ bundled with Java 1.8.0_112.<br>Phospho-RTK Array:Image Lab 6.0.1;HLImage++ 25.0.0<br>Statistical Analysis: GraphPad Prism 9.0.0.

For manuscripts utilizing custom algorithms or software that are central to the research but not yet described in published literature, software must be made available to editors and reviewers. We strongly encourage code deposition in a community repository (e.g. GitHub). See the Nature Portfolio guidelines for submitting code & software for further information.

Data

Policy information about availability of data

All manuscripts must include a data availability statement. This statement should provide the following information, where applicable:

- Accession codes, unique identifiers, or web links for publicly available datasets
- A description of any restrictions on data availability
- For clinical datasets or third party data, please ensure that the statement adheres to our policy

RNA-seq data that support the findings of this study was deposited in the Sequence Read Archive (PRJNA763241, https://www.ncbi.nlm.nih.gov/bioproject/PRJNA763241). Source data are provided with this paper.

Field-specific reporting

Please select the one below that is the best fit for your research. If you are not sure, read the appropriate sections before making your selection.

☒ Life sciences ☐ Behavioural & social sciences ☐ Ecological, evolutionary & environmental sciences

For a reference copy of the document with all sections, see nature.com/documents/nr-reporting-summary-flat.pdf

Life sciences study design

All studies must disclose on these points even when the disclosure is negative.

Sample size

Sample sizes were chosen based on our previous publications, literatures, and power analysis.

Animal experiments on nude mice or NOD-SCID mice, n=8 per group, was based on our previous publications, literatures and power analysis. The power analysis parameters: 1. effect size of 1.67 was assumed on 50% tumor size reduction after 32 days treatment between two groups in comparison and a standard deviation of 30% for tumor volume in each of the comparison groups; 2. 85% power and 5% type I error; 3. two-sample two-tailed t-test for two independent means.

Transgenic mouse model , n=4 in groups with TKI, was base on our previous publications, literatures and power analysis. The power analysis parameters: 1.an approximate average effect size of 2 was observed from nude mice (xenograft) and NOD-SCID mice (PDX); 2. 80% power and 5% type I error; 3. two-sample one-tailed t-test for two independent means.

Before vs After TKI treatment:
The sample size of 10 (before) plus 13 (after) was based on our previous publications. Moreover, 10 is the number of all available TKI-treated NSCLC samples from Jackson(5) or UTSW(5) collected for this study, as the rarity of EGFR mutant and recurrent tumors for re-biopsy.

The power calculation on survival analysis above was performed on http://www.sample-size.net/sample-size-survival-analysis/ .
Other power calculation above was calculated by GPower 3.1 software.

Data exclusions

No collected data were excluded.

Replication

For cell viability, qPCR, and luciferase experiments, the experiment was done in triplicate (3 technical replicates). Data show representative of 3 independent repeats with similar results.
For ELISA assay, Three independent experiments were performed.
Western blot images, tumor images, MRI images, and Immunofluorescence images are representative of three independent experiments(western blot and Immunofluorescence), or indicated number of mouse tumors (tumor images, and MRI images)
RNA-seq data contains 8 conditions, each has three biological independent samples.
All replications above have similar results and are reproducible.
Transcription factor array and Phospho-RTK Array were performed according to the protocol of these commercial kits. No repeated experiments at same conditions were performed. However, experiments were performed on multiple cell lines.

Randomization

For in vivo experiments, the mice were randomly divided into control and different treatment groups, by following steps:

All female 4-6 weeks old nude mice or NOD-SCID mice in the same experiment were ordered, received, injected/implanted, grouped, and treated together. After tumor formation, for randomization, all mice in one experiments would be mix together (all female) and then assigned into different groups, randomly selecting one by one, regardless of tumor sizes, in the sequences like ABBAABBA...(two groups) or ABCDDCBA...(four groups) for the purpose of balancing slow and fast running mice, as fast running mice tend to have a slow growing tumors and hard to grab.

Therefore, the groups in ABCDDCBA... form were well balanced, and allocation of control and different experiment groups can be random.

As the transgenic mice included both sexes, the male(s) could not be merged with other male(s) if not from the same colony since birth, and males could not mixed with females in one cage. Thus, the randomization should be adjusted by those 2 rules above, and each group would contain both male(s) and female(s) after randomly grouping.

Blinding

Animal data: The treatment or control groups were labelled on cage cards. The tumor size measurement every 4 days was set at another time point than treatment. While measuring, the cage cards would be flipped over at first, and length and width of tumor, as well as ear tag ID of

each mouse would be recorded. After an experiment was done and when mice were sacrificed, the mapping from ear tag ID to group ID could be revealed.

MRI: MRI images were analyzed by ImageJ to measure tumor sizes. Mice were labelled by toe and ear clipping. The grouping and drug treatment was performed by one person(KG). The tumor MRI images collection and analysis were performed by 2 persons(KG and GG) without knowing the treatment conditions. The tumor size measurement based on MRI images was performed after all MRI images were collected and mice were sacrificed.

Patient Data: The FFPE tissues have only slide ID, without patients' identifiers. Real-time PCR was performed by KG, patients' medical records were reviewed by AH. After real-time PCR was done, AH would send KG the mapping from slide ID to patients' OS and TKI treatment history. Then the final results were analyzed by KG. TCGA data analysis has no blinding method, all patients meeting the selective critiques described in manuscript would be included in the study.

Reporting for specific materials, systems and methods

We require information from authors about some types of materials, experimental systems and methods used in many studies. Here, indicate whether each material, system or method listed is relevant to your study. If you are not sure if a list item applies to your research, read the appropriate section before selecting a response.

Materials & experimental systems

n/a	Involved in the study	
☐	☒	Antibodies
☐	☒	Eukaryotic cell lines
☒	☐	Palaeontology and archaeology
☐	☒	Animals and other organisms
☐	☒	Human research participants
☒	☐	Clinical data
☒	☐	Dual use research of concern

Methods

n/a	Involved in the study	
☒	☐	ChIP-seq
☒	☐	Flow cytometry
☒	☐	MRI-based neuroimaging

Antibodies

Antibodies used

Target Cat# Vendor(Address) Dilution
EGFR (06-847) EMD Millipore (Billerica, MA) WB1:1000-1:2000
p-EGFR (Tyr1068) (2236) Cell Signaling Technology (Danvers, MA) WB1:1000
p-ERBB2 (Tyr1221/1222) (2243) Cell Signaling Technology (Danvers, MA) WB1:1000
ERBB2 (4290) Cell Signaling Technology (Danvers, MA) WB1:1000
p-ERBB3 (Tyr1289) (4791) Cell Signaling Technology (Danvers, MA) WB1:1000
ERBB3 (12708) Cell Signaling Technology (Danvers, MA) WB1:1000
p-RET (Tyr905) (3221) Cell Signaling Technology (Danvers, MA) WB1:1000
RET (14556) Cell Signaling Technology (Danvers, MA) WB1:1000
YAP (14074) Cell Signaling Technology (Danvers, MA) WB1:1000
STAT3 (9139) Cell Signaling Technology (Danvers, MA) WB1:1000
p-STAT3 (Tyr705) (9145) Cell Signaling Technology (Danvers, MA) WB1:1000
IκBa (4814) Cell Signaling Technology (Danvers, MA) WB1:1000
NIK (4994) Cell Signaling Technology (Danvers, MA) WB1:1000
RELB (10544) Cell Signaling Technology (Danvers, MA) WB1:1000
GR(47411) Cell Signaling Technology (Danvers, MA) WB1:1000
p-GR(4161) Cell Signaling Technology (Danvers, MA) WB1:1000
p-YAP (Tyr357) (ab62751) Abcam (Cambridge, UK) WB1:1000
β-Actin (sc-47778) Santa Cruz Biotechnology (Dallas, TX) WB1:1000
HGF ELISA kit (DHG00B) R&D (Minneapolis, MN)
NRG1-beta1 ELISA kit (EHNRG1) Thermo Fisher (Waltham, MA)
Proteome Profiler Human Phospho-RTK Array (ARY001B) R&D (Minneapolis, MN)

Validation

EGFR (06-847) human,WB
p-EGFR (Tyr1068) (2236) human,WB
p-ERBB2 (Tyr1221/1222) (2243) human,WB
ERBB2 (4290) human,WB
p-ERBB3 (Tyr1289) (4791) human,WB
ERBB3 (12708) human,WB
p-RET (Tyr905) (3221) human,WB
RET (14556) human,WB
YAP (14074) human,WB
STAT3 (9139) human,WB
p-STAT3 (Tyr705) (9145) human,WB
IκBa (4814) human,WB
NIK (4994) human,WB
RELB (10544) human,WB
GR(47411) human,WB

p-GR(4161) human,WB
p-YAP (Tyr357) (ab62751) human,WB
β-Actin (sc-47778) human,WB
HGF ELISA kit (DHG00B) human,ELISA
NRG1-beta1 ELISA kit (EHNRG1) human,ELISA
Proteome Profiler Human Phospho-RTK Array (ARY001B) human Protein-array

Eukaryotic cell lines

Policy information about cell lines

Cell line source(s)

A549: The American Type Culture Collection (ATCC)
A2058: ATCC
OE19: The European Collection of Authenticated Cell Cultures (ECACC), sold by Millipore-Sigma
RT112: DSMZ-German Collection of Microorganisms and Cell Cultures GmbH.
HCC827: Hamon Center for Therapeutic Oncology Research at the University of Texas Southwestern Medical Center
PC9: Hamon Center for Therapeutic Oncology Research at the University of Texas Southwestern Medical Center
H3255: Hamon Center for Therapeutic Oncology Research at the University of Texas Southwestern Medical Center
H441: Hamon Center for Therapeutic Oncology Research at the University of Texas Southwestern Medical Center
H2122: Hamon Center for Therapeutic Oncology Research at the University of Texas Southwestern Medical Center
H1975: Hamon Center for Therapeutic Oncology Research at the University of Texas Southwestern Medical Center
H23: Hamon Center for Therapeutic Oncology Research at the University of Texas Southwestern Medical Center
H1703: Hamon Center for Therapeutic Oncology Research at the University of Texas Southwestern Medical Center
HCC827/ER3: Dr. Trever Bivona, University of California (San Francisco, CA)
HCC827/ER4(ER4A in this manuscript): Dr. Trever Bivona, University of California (San Francisco, CA)
HCC827/ER5: Dr. Trever Bivona, University of California (San Francisco, CA)
HCC827/ER4(ER4B in this manuscript): Dr. Eric Haura, Moffitt Cancer Center (Tampa, FL).

Authentication

ATCC, ECACC, DSMZ does its own authentication on A549, OE19 and RT112, respectively.
Cell lines from Hamon Center for Therapeutic Oncology Research at the University of Texas Southwestern Medical Center were authenticated by DNA fingerprints for cell-line individualization using Promega StemElite ID system, a short tandem repeat (STR)–based assay, at UT Southwestern genomics core.
HCC827 derived lines' authentications were described at:
1. Zhang, Z. et al. Activation of the AXL kinase causes resistance to EGFR-targeted therapy in lung cancer. Nat Genet 44, 852-860 (2012).
2. Yoshida, T. et al. Tyrosine phosphoproteomics identifies both codrivers and cotargeting strategies for T790M-related EGFR-TKI resistance in non-small cell lung cancer. Clin Cancer Res 20, 4059-4074 (2014).

Mycoplasma contamination

Cells were tested negative for mycoplasma contamination using an e-Myco kit (Boca Scientific).

Commonly misidentified lines
(See ICLAC register)

The study did not involve commonly misidentified lines.

Animals and other organisms

Policy information about studies involving animals; ARRIVE guidelines recommended for reporting animal research

Laboratory animals

1. Nude mice (088), female, tumor injection is at 4-6 weeks old, from Charles River Laboratories (Wilmington, MA).
2. NOD SCID mice (394), female, tumor injection is at 4-6 weeks old, from Charles River Laboratories (Wilmington, MA).
3. LSL-Kras G12D mice (008179), tumor induction is at 6-12 weeks old, both sex, breeder Pair, from Jackson laboratories.
4. TetO-EGFR-L858R and CCSP-rtTA mice, tumor induction is at 6-12 weeks old, both sex, gifted from Katerina Politi at Yale University (New Haven, Connecticut, USA)

Wild animals

The study did not involve wild animals.

Field-collected samples

The study did not involve field-collected samples.

Ethics oversight

All animal studies were done under IACUC-approved protocols at UT Southwestern and North Texas VA Medical Center (Dallas, Texas, USA).

Note that full information on the approval of the study protocol must also be provided in the manuscript.

Human research participants

Policy information about studies involving human research participants

Population characteristics

Age, race, gender and other characteristics are not selective critiques, if not indicated as below.

1. Before vs After TKI treatment: FFPE tissues from 23 NSCLC patients (any stages) were obtained from The Jackson Laboratory (10) or UT Southwestern (13). Thirteen patients had no EGFR TKI treatment, and ten patients had undergone any EGFR TKI treatment (gefitinib, erlotinib, afatinib, osimertinib, or dacomitinib).

ID Source Gender Age_at_Dx initial_Bx re_Bx Dx Stage Treatment
UTSW-1 UTSW #NA 59 ex19del #NA AD #NA naïve

UTSW-2 UTSW Female 65 ex19del ex19del AD #NA naïve
UTSW-3 UTSW Female 70 ex19del #NA AD IV naïve
UTSW-4 UTSW Male 64 ex19del #NA AD IA naïve
UTSW-5 UTSW Female 53 L858R T790M AD IV naïve
UTSW-6 UTSW Male 43 L858R #NA AD IIIA naïve
UTSW-7 UTSW Male 66 ex19del #NA AD IB naïve
UTSW-8 UTSW Female 72 ex19del #NA AD #NA naïve
UTSW-9 UTSW Female 65 ex19del ex19del AD #NA EGFR-TKI
UTSW-10 UTSW Male 61 ex19del ex19del AD IB EGFR-TKI
UTSW-11 UTSW Female 50 ex19del #NA AD IV Erlotinib
UTSW-12 UTSW Female 72 ex19del ex19del+T790M AD IV Erlotinib
UTSW-13 UTSW Female 68 ex19del #NA AD IV Erlotinib
TM00193 Jax Female 59 ex19del #NA AD IIIA naïve
TM00186 Jax Female 68 WT #NA AD I naïve
TM00192 Jax Male 57 WT #NA AD IB naïve
TM00231 Jax Male 73 WT #NA AD IB naïve
TM00229 Jax Female 58 WT #NA AD IV naïve
TM00199 Jax Female 44 L858R L858R AD IV Erlotinib
TM00206 Jax Female 57 WT+ELM4-ALK WT+ELM4-ALK AD IV Erlotinib
TM00219 Jax Female 59 ex19del ex19del+T790M AD IV EGFR-TKI Erlotinib
TM00784 Jax Female 42 L858R L858R AD IV EGFR-TKI Erlotinib
TM01244 Jax Female 56 L858R+L62R L858R+L62R AD IV EGFR-TKI Rocileitinib

2. Signatures on Overall Survival (TCGA data): 38 TCGA-LUAD patients (any stages) with classical TKI-sensitive mutations, L858R or exon 19 deletion, but without T790M mutation, were analyzed with their RNAseq and Survival data.

ID Source gender primary_diagnosis tumor_stage Age_at_Dx
TCGA-50-5944 TCGA-LUAD female Acinar cell carcinoma stage ia 69
TCGA-44-2661 TCGA-LUAD female Adenocarcinoma, NOS stage ia 69
TCGA-86-8668 TCGA-LUAD female Bronchio-alveolar carcinoma, mucinous stage ia 61
TCGA-55-6981 TCGA-LUAD female Adenocarcinoma, NOS stage iiia 53
TCGA-J2-8192 TCGA-LUAD female Adenocarcinoma, NOS stage iia 65
TCGA-64-1681 TCGA-LUAD female Adenocarcinoma, NOS stage ia 61
TCGA-49-4490 TCGA-LUAD female Adenocarcinoma, NOS stage iiia 45
TCGA-62-8394 TCGA-LUAD female Adenocarcinoma with mixed subtypes stage iiib 65
TCGA-MP-A4T6 TCGA-LUAD female Adenocarcinoma, NOS stage iiia 76
TCGA-L9-A50W TCGA-LUAD male Adenocarcinoma with mixed subtypes stage iia 75
TCGA-86-8055 TCGA-LUAD male Adenocarcinoma, NOS stage iia 79
TCGA-38-4627 TCGA-LUAD female Adenocarcinoma, NOS stage iia 64
TCGA-55-8206 TCGA-LUAD male Adenocarcinoma, NOS stage ia 56
TCGA-67-6217 TCGA-LUAD female Adenocarcinoma, NOS stage iia 73
TCGA-97-A4M7 TCGA-LUAD male Adenocarcinoma with mixed subtypes stage ia 74
TCGA-97-8171 TCGA-LUAD male Papillary adenocarcinoma, NOS stage iv 81
TCGA-86-8280 TCGA-LUAD female Bronchiolo-alveolar carcinoma, non-mucinous stage iia 54
TCGA-97-8177 TCGA-LUAD female Adenocarcinoma with mixed subtypes stage ib 59
TCGA-55-8096 TCGA-LUAD female Adenocarcinoma, NOS stage ib 67
TCGA-62-8402 TCGA-LUAD female Adenocarcinoma with mixed subtypes stage iiia 73
TCGA-67-3770 TCGA-LUAD female Adenocarcinoma, NOS stage ia 70
TCGA-38-6178 TCGA-LUAD female Adenocarcinoma, NOS stage iiia 70
TCGA-75-6212 TCGA-LUAD female Micropapillary carcinoma, NOS stage iib #NA
TCGA-97-A4M6 TCGA-LUAD female Adenocarcinoma with mixed subtypes stage ia 45
TCGA-97-A4M1 TCGA-LUAD female Adenocarcinoma with mixed subtypes stage ia 52
TCGA-38-4628 TCGA-LUAD female Adenocarcinoma, NOS stage iib 65
TCGA-75-7025 TCGA-LUAD male Adenocarcinoma, NOS stage ib #NA
TCGA-49-4501 TCGA-LUAD female Adenocarcinoma, NOS stage ib 67
TCGA-55-A57B TCGA-LUAD female Adenocarcinoma with mixed subtypes stage ia 80
TCGA-97-8547 TCGA-LUAD female Acinar cell carcinoma stage iiia 78
TCGA-49-4494 TCGA-LUAD male Adenocarcinoma, NOS stage iiia 77
TCGA-MP-A4T9 TCGA-LUAD female Adenocarcinoma, NOS stage iiia 54
TCGA-86-8075 TCGA-LUAD female Adenocarcinoma, NOS stage ib 66
TCGA-71-8520 TCGA-LUAD female Adenocarcinoma, NOS stage ib 60
TCGA-71-6725 TCGA-LUAD female Papillary adenocarcinoma, NOS stage ib 48
TCGA-97-8552 TCGA-LUAD female Bronchiolo-alveolar carcinoma, non-mucinous stage i 55
TCGA-MP-A4SW TCGA-LUAD male Adenocarcinoma, NOS stage iib 53
TCGA-67-3772 TCGA-LUAD female Adenocarcinoma, NOS stage ib 82

Recruitment

1. All available ten TKI-treated NSCLC samples from Jackson (5) or UTSW (5) were collected for this study, as the rarity of EGFR mutant and recurrent tumors for re-biopsy. An almost equal number (13) of TKI-untreated samples were collected from Jackson(5) or UTSW(8). We collected 5+5+5+8=23 samples.

Recruitment criteria:
1a. Patients must consent The Jackson Laboratory or UT Southwestern to use their biopsies for research purposes, under IRB approval.
1b. Either EGFR classical TKI-sensitive mutations(L858R or exon 19 deletion) or EGFR wt. Non-common mutation were excluded as their functions were not very clear. EGFR status unknown were also excluded.
1c. Must be adenocarcinoma. Squamous cell carcinoma (SCC) were excluded, which harbors about 1% EGFR activating

mutation, and SCC patients may not be considered to take TKI treatment.
1d. Must be either recurrent NSCLC tumors collected after EGFR TKI-treated and progressed, or primary NSCLC tumors collected before any treatment.
6 samples were in file(TKI-treated Jackson samples) and 5 were available. There is no further selection, exclusion, or bias.
8 samples were in file(treatment naïve Jackson samples) and 5 were available. There is no further selection, exclusion, or bias.
5 samples were in file(TKI-treated UTSW samples) and 5 were available. There is no further selection, exclusion, or bias.
Hundreds of samples were in file (treatment naïve UTSW samples). For further selection, after each of 5 TKI-treated UTSW cases, we chose the next 2 available treatment naïve cases (1:2), queued by patients' medical record number. Total 10 biopsies' slides were collected. 2 contained no/less RNA. We collected 8 at last.

2. Signatures on Overall Survival (TCGA data): 38 TCGA-LUAD patients (with RNAseq and Survival data available) (any stages) with classical TKI-sensitive mutations, L858R or exon 19 deletion, but without T790M mutation were included in this study.
Recruitment criteria:
2a. Must be in TCGA-LUAD databases. TCGA-LUSC was excluded, because the squamous cell carcinoma harbors about 1% EGFR activating mutation, and TCGA-LUSC patients may not take TKI treatment.
2b. Must have RNAseq data.
2c. Must have DNAseq data(somatic mutation). Must harbor either classical TKI-sensitive EGFR mutations, L858R or exon 19 deletion. Non-common EGFR mutations were excluded, as their functions were not very clear. EGFR wt were excluded, as they normally had no TKI treatment in the present clinical practice.
2d. Must NOT harbor T790M mutation at initial diagnosis, which may case primary resistance.
2e. Must have OS data, from Date-of-Diagnosis to Date of Death (uncensored) or Date of Last Contact (censored).
2f. OS >= 90 days to get TKI treatment of 3 months.
There is no further selection, exclusion, or bias on these 38 cases.

Ethics oversight

Patient tissues and medical records were obtained from UT Southwestern Medical Center and Jackson Laboratory with IRB approval.

Note that full information on the approval of the study protocol must also be provided in the manuscript.
