# Supplementary material for: Comprehensive targeting of resistance to inhibition of RTK signaling pathways by using glucocorticoids
Source: Nat Commun. 2021 Dec 1;12:7014. doi: 10.1038/s41467-021-27276-7 (PMC8636603; doi:10.1038/s41467-021-27276-7)

Uncut WB images for Fig. 1

Gong et al.

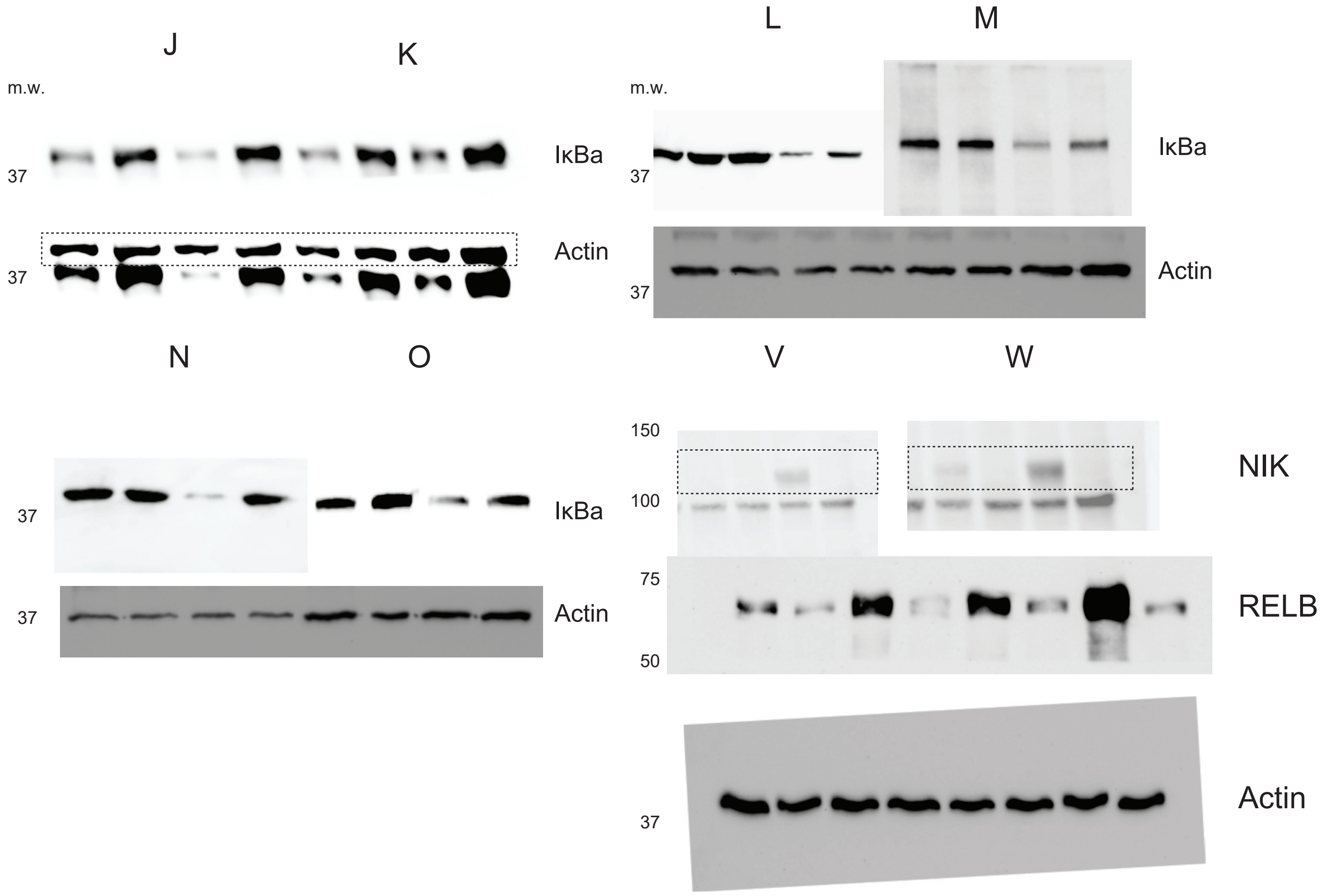

Uncut WB images for Fig.3 (Part of CDEF)

Gong et al.

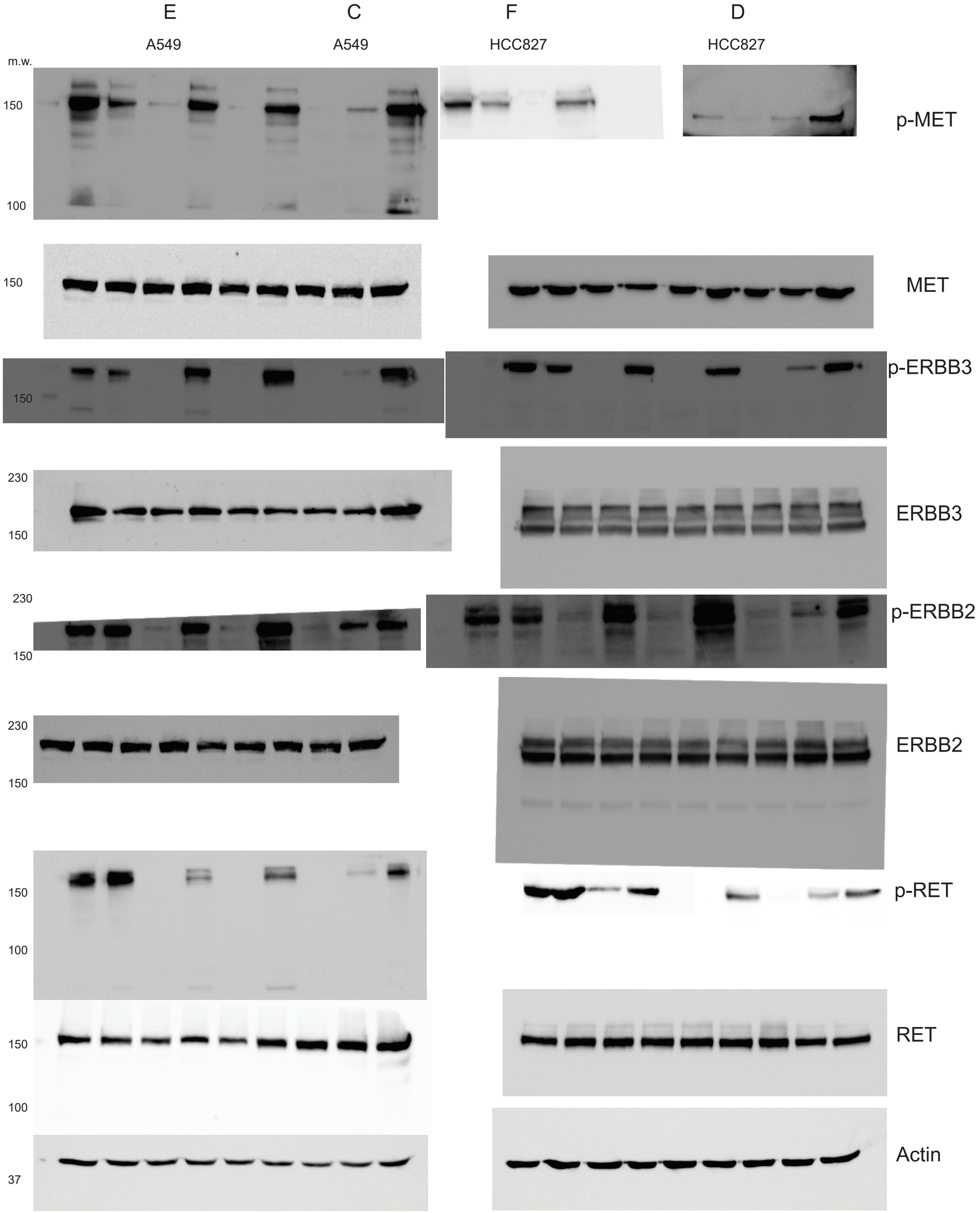

Uncut WB images for Fig. 3(Part of CDEF, and GHI) Gong et al.

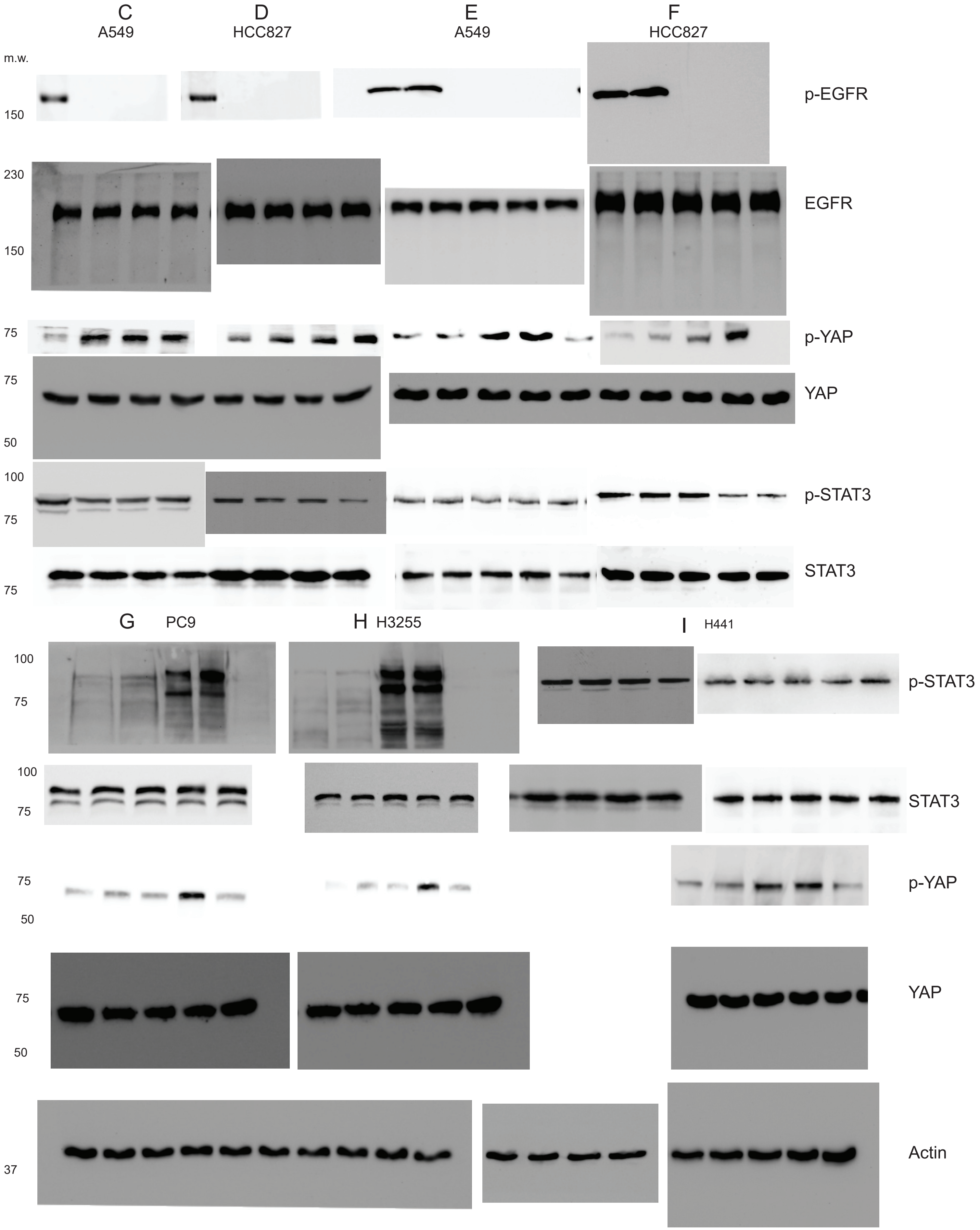

Uncut WB images for Fig. S6

Gong et al.

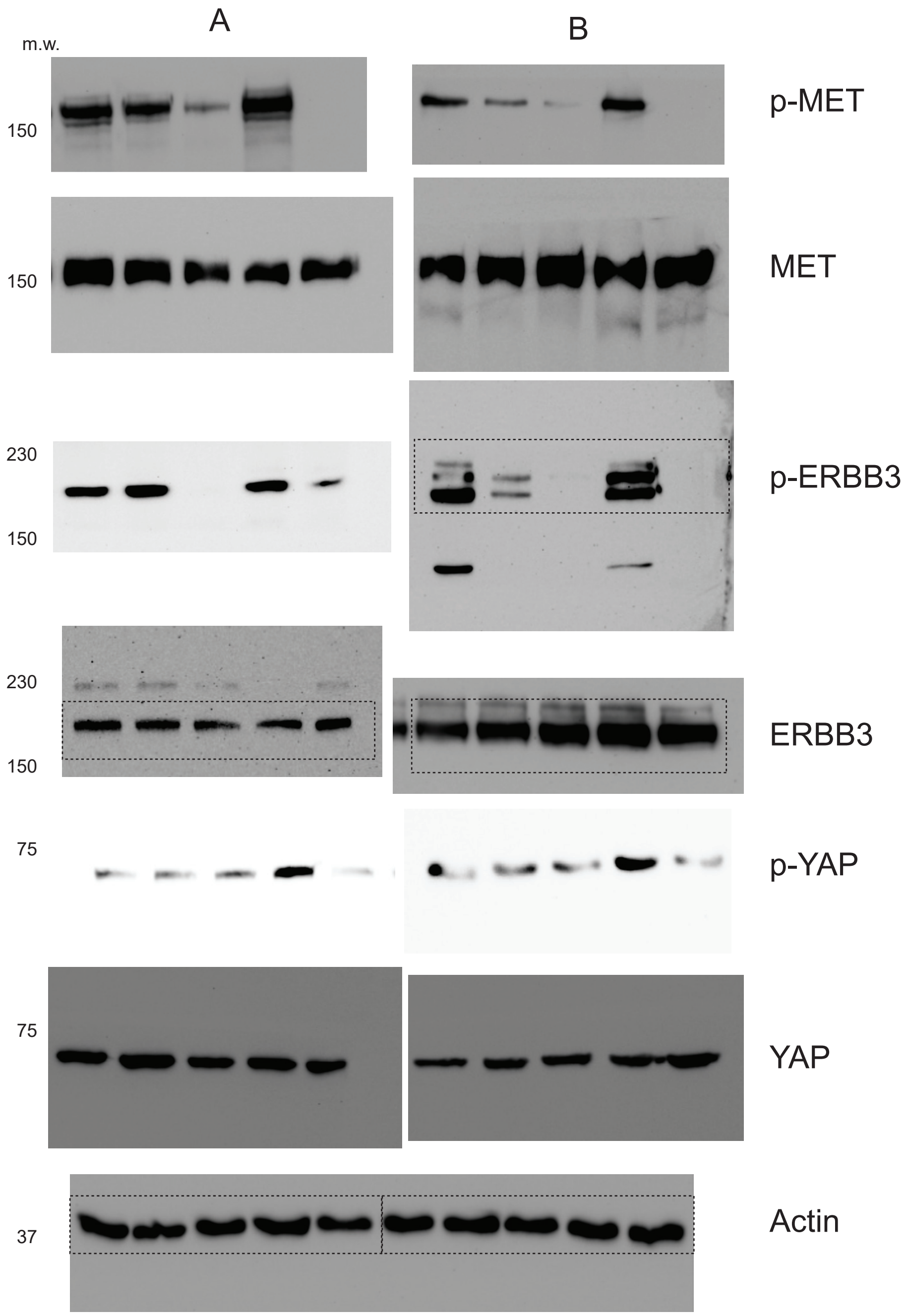

Uncut WB images for Fig. S9

Gong et al.

A

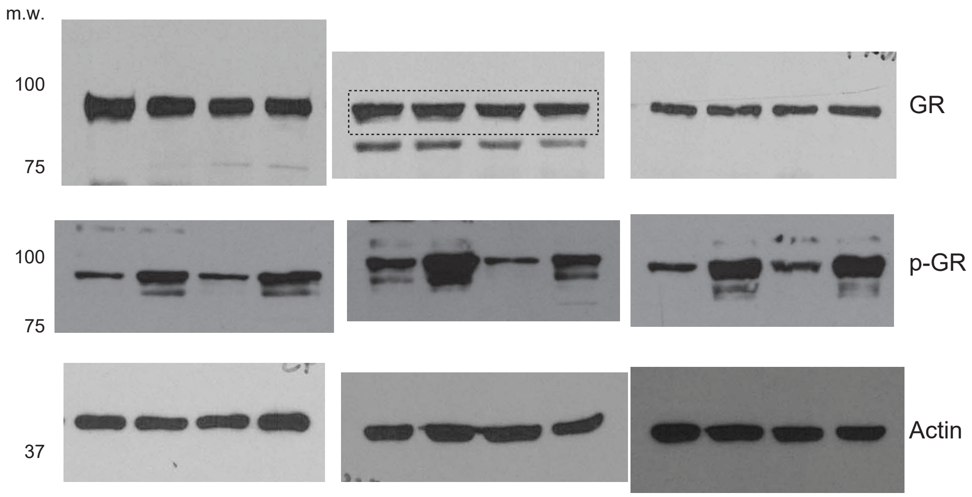

B

C

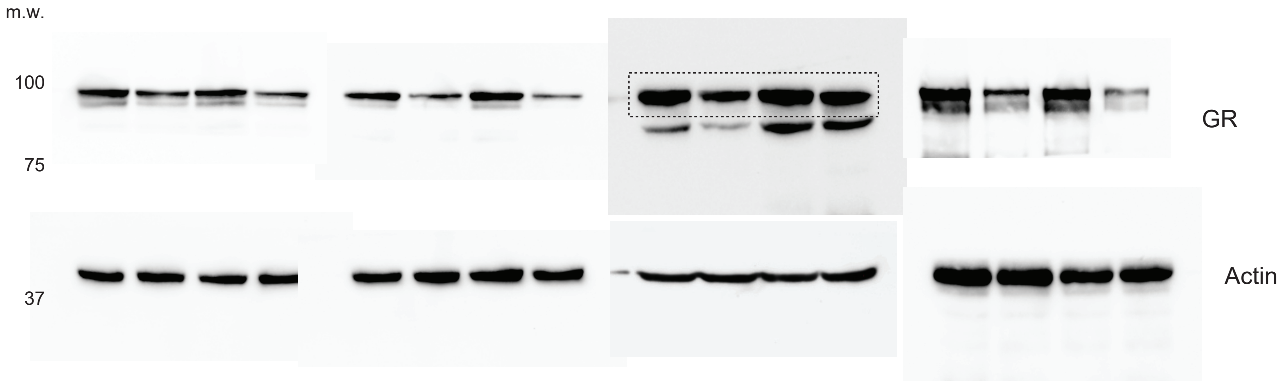

Supplement: Supplementary file 4 — Source Data [file 41467_2021_27276_MOESM4_ESM.zip › uncut WB.pdf]
